# Interplay between MycN and c-Myc regulates radioresistance and cancer stem cell phenotype in neuroblastoma upon glutamine deprivation

**DOI:** 10.7150/thno.42602

**Published:** 2020-05-16

**Authors:** Marion Le Grand, Anna Mukha, Jakob Püschel, Emanuele Valli, Alvin Kamili, Orazio Vittorio, Anna Dubrovska, Maria Kavallaris

**Affiliations:** 1 Children's Cancer Institute, Lowy Cancer Research Centre, UNSW Sydney, NSW, Australia 2052; 2 ARC Centre of Excellence in Convergent Bio-Nano Science and Technology, Australian Centre for Nanomedicine, UNSW Sydney, Australia 2052; 3 School of Women's and Children's Health, Faculty of Medicine, UNSW Sydney, NSW, Australia 2052; 4 OncoRay-National Center for Radiation Research in Oncology, Faculty of Medicine and University Hospital Carl Gustav Carus, Technische Universität Dresden, Helmholtz-Zentrum Dresden-Rossendorf, Dresden, Germany; 5 Helmholtz-Zentrum Dresden - Rossendorf, Institute of Radiooncology - OncoRay, Dresden, Germany; 6 German Cancer Consortium (DKTK), Partner site Dresden, Germany; 7 German Cancer Research Center (DKFZ), Heidelberg, Germany

**Keywords:** Myc members, glutamine metabolism, neuroblastoma, radioresistance, cancer stem cells

## Abstract

Targeting glutamine metabolism has emerged as a potential therapeutic strategy for Myc overexpressing cancer cells. Myc proteins contribute to an aggressive neuroblastoma phenotype. Radiotherapy is one of the treatment modalities for high-risk neuroblastoma patients. Herein, we investigated the effect of glutamine deprivation in combination with irradiation in neuroblastoma cells representative of high-risk disease and studied the role of Myc member interplay in regulating neuroblastoma cell radioresistance.

**Methods**: Cell proliferation and viability assays were used to establish the effect of glutamine deprivation in neuroblastoma cells expressing c-Myc or MycN. Gene silencing and overexpression were used to modulate the expression of Myc genes to determine their role in neuroblastoma radioresistance. qPCR and western blot investigated interplay between expression of Myc members. The impact of glutamine deprivation on cell response following irradiation was explored using a radiobiological 3D colony assay. DNA repair gene pathways as well as CSC-related genes were studied by qPCR array. Reactive Oxygen Species (ROS) and glutathione (GSH) levels were detected by fluorescence and luminescence probes respectively. Cancer-stem cell (CSC) properties were investigated by sphere-forming assay and flow cytometry to quantify CSC markers. Expression of DNA repair genes and CSC-related genes was analysed by mining publicly available patient datasets.

**Results**: Our results showed that glutamine deprivation decreased neuroblastoma cell proliferation and viability and modulated Myc member expression. We then demonstrated for the first time that combined glutamine deprivation with irradiation led to a selective radioresistance of *MYCN*-amplified neuroblastoma cells. By exploring the underlying mechanism of neuroblastoma radioresistance properties, our results highlight interplay between c-Myc and MycN expression suggesting compensatory mechanisms in Myc proteins leading to radioresistance in *MYCN*-amplified cells. This result was associated with the ability of *MYCN*-amplified cells to dysregulate the DNA repair gene pathway, maintain GSH and ROS levels and to increase the CSC-like population and properties. Conversely, glutamine deprivation led to radiosensitization in non-*MYCN* amplified cell lines through a disruption of the cell redox balance and a trend to decrease in the CSC-like populations. Mining publicly available gene expression dataset obtained from pediatric neuroblastoma patients, we identified a correlation pattern between Myc members and CSC-related genes as well as a specific group of DNA repair gene pathways.

**Conclusions**: This study demonstrated that MycN and c-Myc tightly cooperate in regulation of the neuroblastoma CSC phenotypes and radioresistance upon glutamine deprivation. Pharmacologically, strategies targeting glutamine metabolism may prove beneficial in Myc-driven tumors. Consideration of MycN/c-Myc status in selecting neuroblastoma patients for glutamine metabolism treatment will be important to avoid potential radioresistance.

## Introduction

Neuroblastoma is an enigmatic, multifaceted tumor of the peripheral nervous system that represents the most common solid tumor in children under five years of age 1. Based on its cellular and biological heterogeneity, neuroblastoma behavior can range from low-risk cancers with a tendency toward spontaneous regression, to high-risk ones with extensive growth, early metastasis and a poor prognosis 2, 3.

Members of the Myc family of transcription factors (c-Myc, MycN and L-Myc) play critical roles in many cellular processes required for tumorigenesis including cell growth and differentiation, metabolism, and genome stability 4. High expression levels of Myc members contribute to aggressive neuroblastoma phenotypes 5. The amplification of the *MYCN* oncogene, which occurs in 25% of neuroblastoma patients and 40% of high-risk cases, currently remains the best-characterized poor prognostic genetic marker of this disease 3, 5. Alternatively, elevated c-Myc expression correlates with poor prognosis in *MYCN* non-amplified neuroblastoma 6.

Radiation therapy is one of the mainstays of treatment for high-risk neuroblastoma 7. The risk of relapse still presents a significant challenge and optimal application of radiation to high-risk patients remains elusive. Tumor relapse after radiotherapy has been attributed to cancer stem cells (CSCs) 8-10. CSCs are defined as a subpopulation within a tumor that can self-renew, are highly tumorigenic and are resistant to conventional chemo- and radiotherapy 11, 12. Several studies have demonstrated that neuroblastoma contains a cell population having stem-cell like properties with enhanced expression of CSC markers including CD117, CD133, OCT4 and ALDH activity attributed to the expression of ALDH1A2 and ALDH1A3 proteins 13-16. There is increasing evidence that Myc members play specific roles in CSCs. It has been shown that Myc-induced epigenetic reprogramming enhances the CSC phenotypes 17. Furthermore, CSCs can alter their metabolism by increasing glycolysis and glutaminolysis through Myc member expression to maintain their proliferation rate 18.

Metabolism in cancer cells is fundamentally altered and is now established as a hallmark of cancer development 19. As cancer cells rapidly proliferate, metabolism must be altered to sustain adequate macromolecule biosynthesis, energy production and redox balance 20. The importance of glutamine as a global and critical nutrient in cancer cells has become better understood and appreciated 21. Glutamine metabolism plays essential roles in cancer cell survival and proliferation by supplying metabolite pathways. Moreover, by maintaining redox balance through synthesis of glutathione, glutamine metabolism contributes to radiotherapy and chemotherapy resistance by protecting tumor cells against oxidative stress 21. Myc transcription factors are considered as the main oncoproteins responsible for glutamine addiction of tumor cells 22. c-Myc drives glutamine uptake and catabolism by activating the expression of genes involved in glutamine metabolism, including glutaminase, *GLS* and *SLC1A5* (solute carrier family 1 (neutral amino acid transporter), member 5) 23, 24. In *MYCN*-amplified neuroblastoma, cells predominantly rely on activation of ASCT2 (solute carrier family 1 member 5, SLC1A5) to maintain sufficient levels of glutamine essential for the TCA cycle anaplerosis 25, 26. As such, Myc-driven tumors are particularly dependent on glutamine metabolism to sustain their viability and targeting glutamine metabolism has emerged as a potential therapeutic strategy for Myc overexpressing cancer cells 21.

Combining radiotherapy with a strategy that targets glutamine metabolism might be beneficial for treatment refinement. In this study, we investigated the role of Myc family-driven glutamine metabolism on neuroblastoma radioresistance properties. Our results demonstrate that MycN and c-Myc tightly cooperate in regulation of the CSC phenotype and radioresistance properties in neuroblastoma cells upon glutamine deprivation.

## Materials and Methods

### Cell culture

SK-N-AS (RRID:CVCL_1700), SH-EP (RRID:CVCL_0524) and Kelly (RRID:CVCL_2092) were obtained from European Collection of Cell Cultures. SK-N-BE(2)-C (referred as BE(2)-C, RRID:CVCL_0529) and SH-SY5Y (RRID:CVCL_0019) were provided by Dr. June Biedler, Memorial Sloan-Kettering Cancer Center, (New York, NY). Upon receipt, cell master stocks were prepared and cells for experiments were passaged for less than 3 months. Master stock cells were validated by PCR (STR profiling) by Cell Bank Australia or Garvan Institute (Sydney, Australia). Human SH-EP/TET21N cells, derived from SH-EP neuroblastoma cells, express *MYCN* upon the control of tetracycline (Tet-off) 27 and was kindly provided by Dr. M. Schwab from the German Cancer Research Center (Heidelberg, Germany). SK-N-AS and SH-SY5Y pCDH and pMYCN cells were derived from SK-N-AS and SH-SY5Y cells respectively by stable transfection with *MYCN*-expression vector (pCDH-CMV-MCS-EF1-copGFP-T2A-Puro, System Biosciences, CA, USA). Neuroblastoma cell lines were cultured in DMEM or RPMI-1640 media (Life Technologies) supplemented with 10% FBS (#F0392; Sigma-Aldrich, GE) and with or without 2 mmol/L L-glutamine (Invitrogen). Cells were cultured at 37°C and 5% CO_2._ All cell lines were regularly screened to ensure the absence of mycoplasma contamination using the MycoAlert MycoPlasma Detection Kit (Lonza, Switzerland). Cells were genotyped using microsatellite polymorphism analyses directly prior to experimentation.

### Drugs and Reagents

Stock solutions of topotecan (Sigma-Aldrich, #E1383), CB-839 (Sigma-Aldrich, #1439399) and Prexasertib (Biomol, Cayman Chemical, #21490) were prepared in dimethyl sulfoxide (DMSO). Stock solutions were kept at -20°C and were freshly diluted in the culture medium for experiments.

### Growth inhibition assay

For all cell lines, the number of cells seeded (from 2,500 to 3,000 cells per well, depending on the cell line) in 96-well plates was first optimized to ensure sustained exponential growth for 4-6 days. For all cell lines, cells were treated 24 h after seeding with full media with a range of CB-839 concentrations. After 72 h drug incubation, metabolic activity was detected by addition of Alamar blue by spectrophotometric analysis. Cells were treated 24 h after seeding with full media or glutamine-deprived media. After 48 h, media were changed and cells were treated with a range of topotecan concentrations in full media. After 24 h drug incubation, metabolic activity was detected by addition of Alamar blue by spectrophotometric analysis. Cell proliferation was determined and expressed as a percentage of untreated control cells. The determination of IC_50_ values was performed using GraphPad Prism software (GraphPad Software Inc, La Jolla, CA).

### Pharmacological inhibition of Chk1

Cells were seeded in 6-well plates (400,000 cells/well) in complete growth medium. 24 h after seeding medium was changed to complete medium containing 50 nM Prexasertib. DMSO was used as a control. Cells were incubated with Prexasertib or DMSO for 48 h and then harvested and used for Western blotting and RNA isolation.

### Trypan blue dye exclusion assay

Cells were cultured in 6-well plates. 24 h after seeding, cells were washed by PBS and fresh media with or without glutamine was added. Cell number and viability were quantified by Trypan blue staining and cell counting at indicated time points.

### 3D colony forming assay

Following 24 h or 48 h of glutamine deprivation, cells were plated in 96-well plates in 5 technical replicates at a density of 1,000-2,000 cells/well depending on the cell lines with medium containing 20% Matrigel (#FAL356230; BD Biosciences). 24 h after plating, cells were irradiated with doses of 2, 4, 6 and 8 Gy of 200 kV X-rays (Yxlon Y.TU 320; 200 kV X-rays, dose rate 1.3Gy/min at 20 mA; 0.5 mm Cu filter) or X-RAD320; Precision X-ray irradiation (Connecticut, USA). The plates were incubated in a humidified 37°C incubator supplemented with 5% CO_2_. After 7 to 14 days of culturing depending on the cell lines, the colonies were counted. Plating efficacies and surviving fractions were calculated as described previously 28. Regression analysis with SPSS software (IBM, USA) was used; α and β values obtained from these calculations were used to build survival curves.

### Flow cytometry analysis

Aldehyde dehydrogenase activity was analysed using ALDEFLUOR assay kit (Stem Cell Technologies, #01700) according to the manufacturer's protocol. Cells were collected as a single-cell suspension and resuspended in flow cytometry buffer (500 mL PBS, 3% FBS, 25 mM HEPES, 1 mM EDTA) or ALDEFLUOR buffer (supplemented). For the analysis of the percentage of CD133-positive population cells were stained with CD133-PE (Miltenyi Biotec, #5181106100, clone 293C3, dilution 1:50) antibodies. As negative controls we used cells treated with DEAB (for ALDEFLUOR assay; #01705) or with isotype IgG (Miltenyi Biotec, mouse IgG2b-PE #5181106018, dilution 1:50). Samples were analysed with the BD LSRII and BD Celesta flow cytometers (Beckton Dickinson, USA). A minimum of 100,000 viable cell events was recorded per sample. Data were analysed using FlowJo software (version 7.6.2) and gates were set according to the individual isotype controls (for CD133-PE-stained cells) or DEAB-treated cells (for ALDEFLUOR assay).

### Sphere forming assay

Cells were plated as single cell suspension at a density of 1,000-2,000 cells depending on the cell lines in 24 well ultra-low attachment plates (Corning) in Mammary Epithelial Basal Medium (MEBM) (Lonza, Germany, #CC-3151) supplemented with 4 μg/ml insulin (Sigma-Aldrich, #91077C-250MG), B27 (Invitrogen, # 17504044), 20 ng/mL epidermal growth factor (EGF) (Peprotech, #AF-100-15), 20 ng/ml basic fibroblast growth factor (FGF) (Peprotech, #100-18B) and 1 mM L-glutamine (Sigma-Aldrich, G7513-100ML). 24 h after plating cells were irradiated with 4 Gy X-rays or sham-irradiated and left to grow at 37°C and 5% CO_2._ Spheres were analysed 14 days after cell plating. Plates were automatically scanned using the Celigo S Imaging Cell Cytometer (Brooks). The number of spheres was analysed using ImageJ software. Cell aggregates were discriminated from spheres based on their shape, size and structure and excluded from analysis.

### siRNA transfection

For knockdown of *MYCN* and *c-MYC* expression, cells were transfected with RNAiMAX (Life Technologies GmbH, #13778075) according to the manufacturer's protocol. siRNAs used were scrambled (#SI03650318 , Qiagen, and #15-7826-4/4, Eurofins Genomics), *MYCN* (sequence #5 - #SI03078222; sequence #6 - #SI03087518; sequence #3 - #SI00076300; Qiagen) and *c-MYC* (sequence #1 - MYC-106821, #35-6922-15/15, and sequence #2 - MYC-3107, #35-6922-13/15, Eurofins Genomics). For knockdown of *c-MYC* expression with pooled siRNA SMARTpool (#L-003282-02-0005, Dharmacon) was used; for knockdown of *MYCN* expression with pooled siRNA a mixture of *MYCN* sequences #5, #6 and #3 was used. Nucleotide sequences are listed in Table S1. Cell transfected with scrambled siRNA were used as control. The concentration of single siRNAs was 40 pmol/well. When knockdown of both c-Myc and MycN was performed, the concentration of each single siRNA remained the same, while the amount of scrambled siRNA was doubled.

### mRNA extraction, cDNA synthesis and qPCR analysis

Expression of genes at mRNA level was analysed by real-time qPCR. Total RNA was isolated from cells using RNeasy Plus Mini Kit (Qiagen, #74134) according to manufacturer's protocol. cDNA synthesis was performed with PrimeScript RT Reagent Kit (Takara, #RR037A) according to manufacturer's protocol. qPCR was done with TB Green Premix Ex Taq II Kit (Takara, #RR820L) using StepOnePlus Real-Time PCR System (Applied Biosystems). DeltaCT values of each reaction were normalized to the deltaCT values of the ACTB gene. Oligonucleotide sequences are listed in Table S1. Expression levels of 84 DNA repair genes were analysed using RT² Profiler PCR Array Human DNA Repair (Qiagen, #PAHS-042Z) according to the manufacturer's recommendations. Expression of 84 CSC-related genes was analysed by RT² Profiler PCR Array Human Cancer Stem Cells (Qiagen, #PAHS-176Z) according to the manufacturer's recommendations. For RT^2^ Profiler Array experiments the equal quantities of RNA from two independent repeats were pooled. The evaluation of relative expression levels for *PROM1* upon *c-MYC* or *MYCN* knockdown was done using pooled samples prepared from three independent repeats. The knockdown of *c-MYC* and *MYCN* was validated in each sample prior to pooling.

### Western blot analysis

Western blot assays were performed as previously described 29. The primary antibodies used are listed in Table S1. Peroxidase-conjugated secondary antibodies and ECL Plus Western Blotting Detection Reagent (Thermo Fisher Scientific, Victoria, Australia) were used for visualization and signal quantification was done with Image J software.

### *In vivo* study

Tumor sections were obtained from BE(2)-C and SH-SY5Y xenograft previously performed in our lab. Animal experiments were approved by the Animal Ethics Committee, University of New South Wales (ACEC #16/54B and 17/53B). Female BALB/c nude mice (6-8 weeks old) were obtained from the Australian Bio Resources Facility (Moss Vale, New South Wales, Australia). Cells (1x10^6^ BE(2)-C or SH-SY5Y) were suspended in 50 μL PBS / 50 μL matrigel (Corning, NY) and were inoculated subcutaneously into the flanks of the nude mice. Twice a week, tumor volumes were determined by caliper measurements, according to the formula length × width × depth ×0.5236. Mice were sacrificed when their tumor volume exceeded 1,000 or 1,500 mm3. Tumor sections were obtained from control BE(2)-C and SH-SY5Y cells xenograft.

### Determination of glutathione and reactive oxygen species levels

Following 24 h glutamine deprivation, cells were irradiated with 4 Gy X-rays or sham-irradiated and analysis performed 24 h later. Total glutathione (GSH) levels were tested using intracellular GSH-Glo glutathione assay kit (Promega, #V6911) according to manufacturer's protocol. Data were expressed in Relative Light Unit (RLU) normalised to protein concentration. Treatment with buthionine sulfoximine (BSO; Sigma-Aldrich; #B-2515) was used as positive control. Total reactive oxygen species (ROS) levels were tested using dihydroethidium (DHE) assay kit (Abcam, #ab236206) according to manufacturer's protocol. Data were expressed in Relative Fluorescent Unit (RFU) normalised to cell number. Treatment with Antimycin A and N-acetyl cysteine (NAC) were used as positive and negative controls respectively. Luminescence and fluorescence were measured with a Victor plate reader (Perkin Elmer, Melbourne, VIC, Australia).

### Expression analysis on neuroblastoma tumor cohorts

Analysis of mRNA expression in tumor samples of neuroblastoma patients was done by the built-in tools of cBioPortal (www.cbioportal.org) and by SUMO software (http://angiogenesis.dkfz.de/oncoexpress/software/sumo/). RefSeq mRNA expression data was extracted from the cohort of 1,089 patients (pediatric neuroblastoma TARGET study, 2018). The co-expression analyses were performed in cBioPortal built-in tools, and Spearman correlation coefficients were used to build the plots. The correlation maps were generated in SUMO using Pearson correlation coefficients.

### Statistical analysis

Each experiment was performed at least in triplicate. Data are presented as mean ± S.E.M. Statistical significance was tested using unpaired Student's t test. For experiments using multiple variables, statistical significance was assessed *via* two-way ANOVA. A significant difference between two conditions was recorded for *p < 0.05; **p < 0.01; ***p < 0.001.

## Results

### Glutamine deprivation decreases cell number and viability in neuroblastoma cells

**Figure 1:**
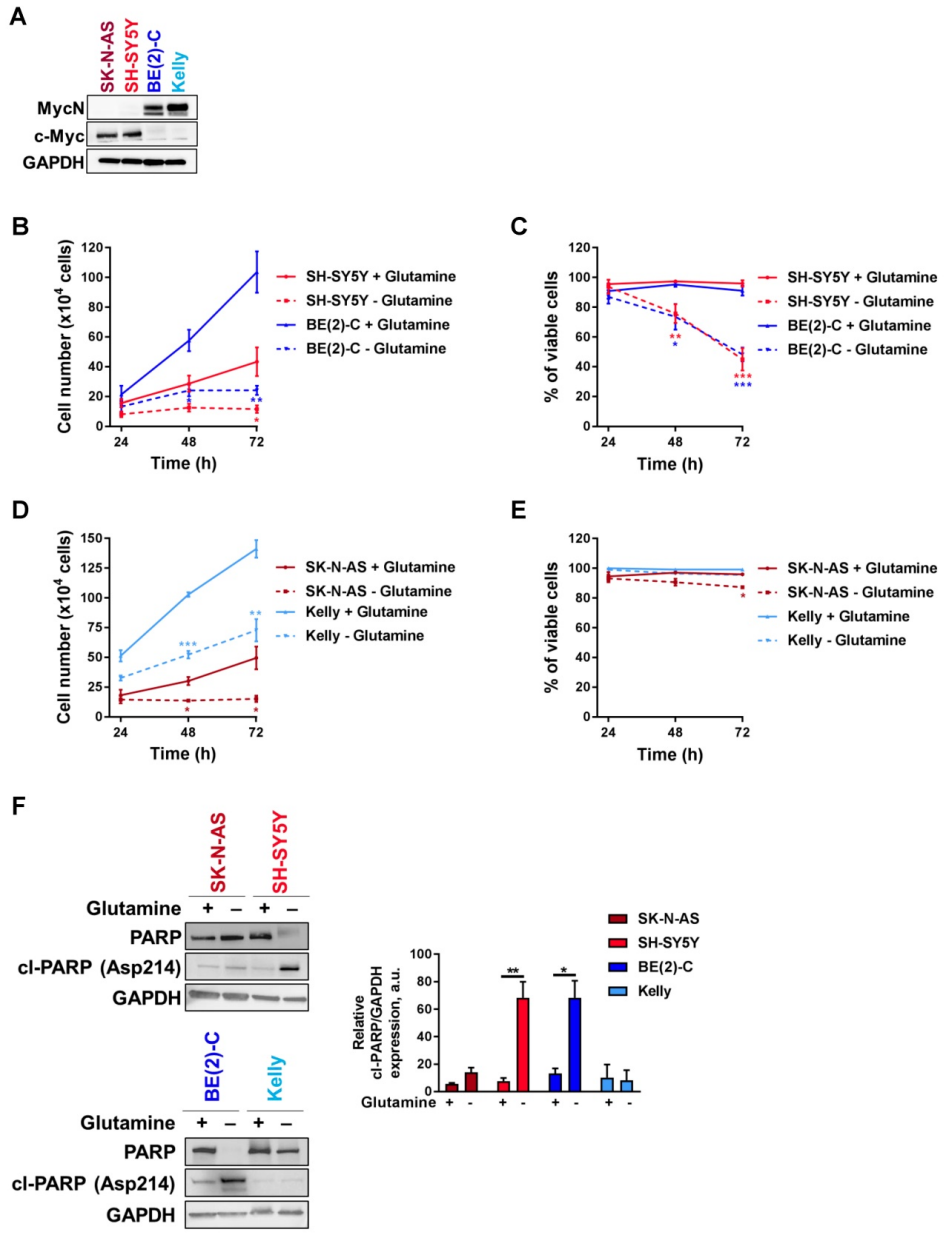
** Glutamine deprivation decreases cell number and viability in neuroblastoma cells.** (A) Western blot analysis of Myc member expression in protein extracts from a panel of four neuroblastoma cell lines. GAPDH was used as a loading control. (B, D) Cell number and (C, E) cell viability performed using Trypan Blue Dye exclusion assay on neuroblastoma cells cultured with or without glutamine from 24 to 72 h. Data are reported as averages (n ≥ 3; ± S.E.M; *p < 0.05; **p < 0.01; ***p < 0.001). (F) Analysis of PARP and cleaved-PARP levels by western blotting in a panel of neuroblastoma cells 72 h upon glutamine deprivation. GAPDH was used as a loading control. Graph showing the quantitative analysis of relative cleaved-PARP protein expression after normalizing to GAPDH. (n = 3; ± S.E.M; *p < 0.05; **p < 0.01).

Tumors with enhanced expression of Myc member oncogenes are particularly dependent on glutamine metabolism to sustain their viability 21. To examine the importance of Myc member expression to glutamine deprivation conditions, four cell lines representing high-risk neuroblastoma disease, two with high c-Myc expression (SK-N-AS and SH-SY5Y) and two with *MYCN* amplification (BE(2)-C and Kelly) (Figure 1A), were cultured in glutamine-free media and their proliferation rate and viability were assessed periodically. BE(2)-C and SH-SY5Y cells showed reduced cell number and viability compared to cells cultured in normal growth media (Figures 1B, C). Indeed, cell number was significantly decreased after 48 h of exposure to glutamine-free conditions, and this was maintained up to the end point of the experiment (72 h; p < 0.05; Figure 1B). The cell viability was also significantly reduced in SH-SY5Y by 53 ± 4% (p < 0.001, Figure 1C) and in BE(2)-C by 48 ± 2.5% (p < 0.001, Figure 1C). Although the same trend of a decrease in the cell number was shown in SK-N-AS and Kelly (Figure 1D), their cell viability was not affected by glutamine deprivation (Figure 1E). To reinforce these results, CB-839 a well-known glutamine inhibitor was tested in both SH-SY5Y and BE(2)-C cells. Our results showed that CB-839 had a dose-dependent effect on cell viability in both cell lines (Figure S1A). To better understand the effect on glutamine deprivation in neuroblastoma, the apoptotic marker cleaved-PARP was analysed by western blot. Glutamine deprivation conditions did show a significant increase in cleaved-PARP only in SH-SY5Y and BE(2)-C neuroblastoma cell lines, while apoptosis induction was not significantly induced in SK-N-AS and Kelly cells (Figure 1F). To further investigate the potential link between the level of *MYCN* expression and cell response to glutamine deprivation, we utilized as a model the SH-EP/TET21N human neuroblastoma cell line, in which *MYCN* expression can be repressed by doxycycline, and two non-*MYCN*-amplified neuroblastoma cell lines, SH-SY5Y and SK-N-AS, which were stably overexpressing *MYCN* (Figure S1B). All cell lines tested showed a reduction in proliferation rate compared to cells cultured in normal growth media, while only SH-SY5Y and SH-EP/TET21N had reduced cell viability (Figure S1C-H; comparison between full and dotted lines). We found that overexpression of MycN significantly reduced cell number in SK-N-AS upon glutamine deprivation (Figure S1C, comparison between dotted lines), while no significant effect was observed in SH-SY5Y and SH-EP/TET21N (Figures S1E, G, comparison between dotted lines). Moreover, no change in the cell viability was shown in SK-N-AS and SH-SY5Y overexpressing MycN cells (Figures S1D, F, comparison between dotted lines), whereas a significant decrease in the SH-EP/TET21N cell viability was observed (Figure S1H, comparison between dotted lines). Altogether, these results illustrate that glutamine deprivation could affect both the cell number and viability of high-risk neuroblastoma cells in a cell-line dependent manner.

### Interplay between expression of Myc members in neuroblastoma cells upon glutamine deprivation

**Figure 2:**
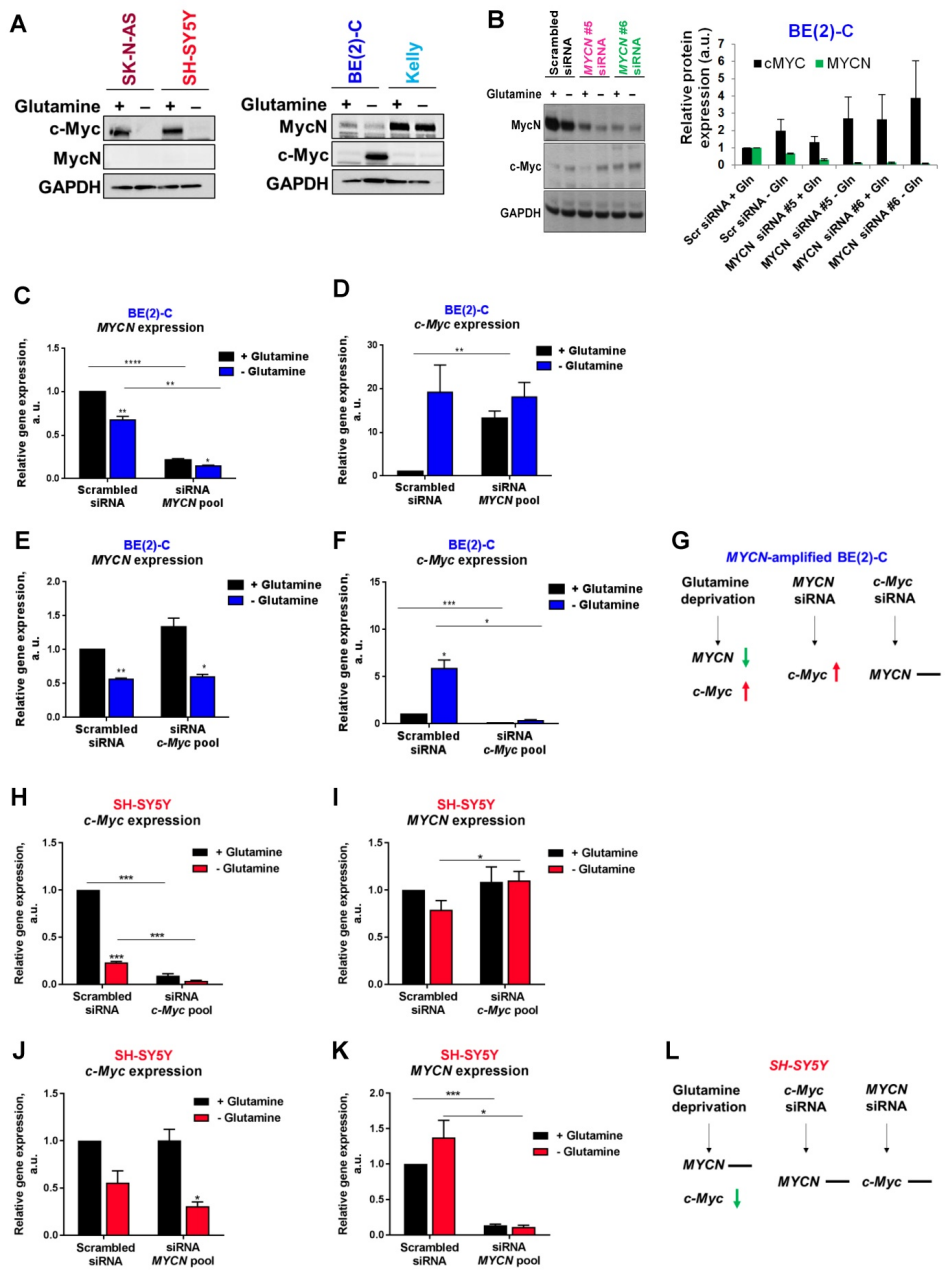
** Interplay between Myc member expression in neuroblastoma cells upon glutamine deprivation.** (A) Western blot analysis of Myc member expression in neuroblastoma cells cultured 24 h with or without glutamine. GAPDH was used as a loading control. (B) Analysis of MycN and c-Myc levels by western blotting in BE(2)-C cells following *MYCN* downregulation upon glutamine deprivation. GAPDH was used as a loading control. Graph showing the quantitative analysis of relative MycN and c-Myc protein expression after normalizing to GAPDH. (n = 4; ± S.E.M). (C, D) Relative mRNA expression of (C) *MYCN* and (D) *c-MYC* following *MYCN* downregulation upon glutamine deprivation in BE(2)-C cells. (E, F) Relative mRNA expression of (E) *MYCN* and (F) *c-MYC* following *c-MYC* downregulation upon glutamine deprivation in BE(2)-C cells. (G) Schematic representation of the effect of glutamine deprivation, *MYCN* or *c-MYC* siRNA on mRNA *MYC* member expression level in BE(2)-C cells. (H, I) Relative mRNA expression of (H) *c-MYC* and (I) *MYCN* following *c-MYC* downregulation upon glutamine deprivation in SH-SY5Y cells. (J, K) Relative mRNA expression of (J) *c-MYC* and (K) *MYCN* following *MYCN* downregulation upon glutamine deprivation in SH-SY5Y cells. (L) Schematic representation of the effect of glutamine deprivation, *MYCN* or *c-MYC* siRNA on mRNA *MYC* member expression level in SH-SY5Y cells. (n ≥ 3; ± S.E.M; *p < 0.05; **p < 0.01; ***p < 0.001).

To evaluate if MycN and c-Myc expression could be affected by glutamine deprivation, analysis of protein levels by western blotting was first performed. Our results showed that 24 h glutamine deprivation reduced c-Myc expression in SK-N-AS and SH-SY5Y cells without impacting MycN expression (Figure 2A). In the *MYCN*-amplified BE(2)-C cells, our results showed a decrease in MycN expression and a concomitant increase in c-Myc expression upon glutamine deprivation, whereas no effect on expression of MycN and c-Myc was observed in Kelly cells. To better understand the relationship of Myc members upon glutamine deprivation, we then performed siRNA-mediated knockdown of *MYCN* in BE(2)-C cells and analysed Myc member mRNA and protein expression. Glutamine deprivation or knockdown of *MYCN* by gene silencing led to an up-regulation of *c-MYC* mRNA and c-Myc protein levels in the BE(2)-C cells (Figures 2B-D). In contrast, siRNA mediated knockdown of *c-MYC* expression did not impact *MYCN* expression, suggesting that this feedback mechanism is not bidirectional (Figures 2E-G). The same experiments were performed in the c-Myc expressing SH-SY5Y cells. Glutamine deprivation strongly decreased *c-MYC* mRNA levels (Figure 2H) without significantly modifying *MYCN* mRNA levels (Figure 2I). Moreover, no interplay was shown between *MYCN* and *c-MYC* at the mRNA levels using both siRNAs targeting *MYCN* or *c-MYC* (Figures 2H-L). However, glutamine deprivation combined with *c-MYC* gene knockdown led to a small but significant increase in *MYCN* mRNA level in SH-SY5Y cells (Figure 2I; p < 0.05). Collectively, our data suggest interplay between *MYCN* and *c-MYC* expression in *MYCN*-amplified neuroblastoma cells upon glutamine deprivation.

### Glutamine deprivation alters neuroblastoma radioresistant properties depending on Myc member expression

**Figure 3:**
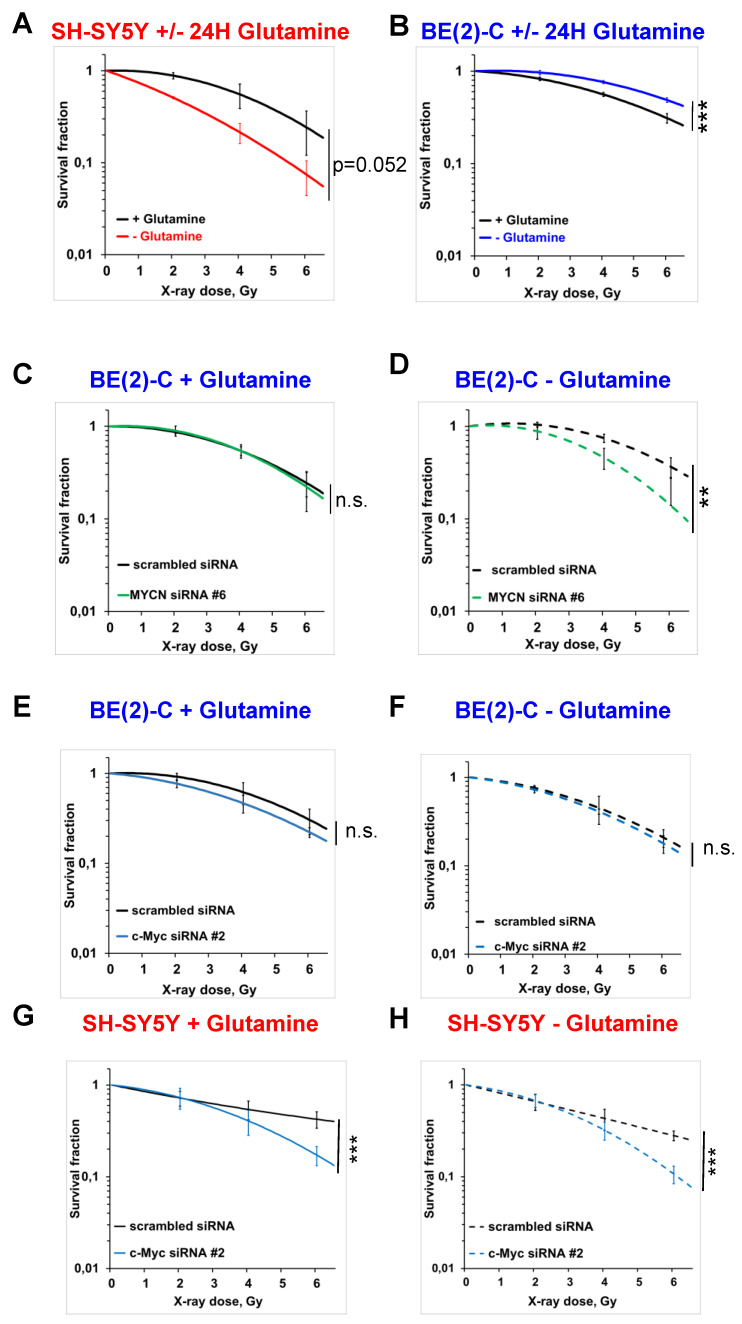
** Glutamine deprivation alters neuroblastoma radioresistant properties.** (A, B) Radiobiological colony forming assay of (A) SH-SY5Y and (B) BE(2)-C cells upon 24 h glutamine deprivation (n ≥ 3; ± S.E.M; ***p < 0.001). (C, D) Radiobiological colony forming assay of BE(2)-C cells following *MYCN* downregulation (C) with glutamine or (D) upon 24 h glutamine deprivation (n ≥ 3; ± S.E.M; ns p > 0.05; **p < 0.01). (E, F) Radiobiological colony forming assay of BE(2)-C following *c-MYC* downregulation (E) with glutamine or (F) upon 24 h glutamine deprivation (n ≥ 3; ± S.E.M; ns p > 0.05). (G, H) Radiobiological colony forming assay of SH-SY5Y following *c-MYC* downregulation (G) with glutamine or (H) upon 24 h glutamine deprivation (n ≥ 3; ± S.E.M; ***p < 0.001).

**Figure 4:**
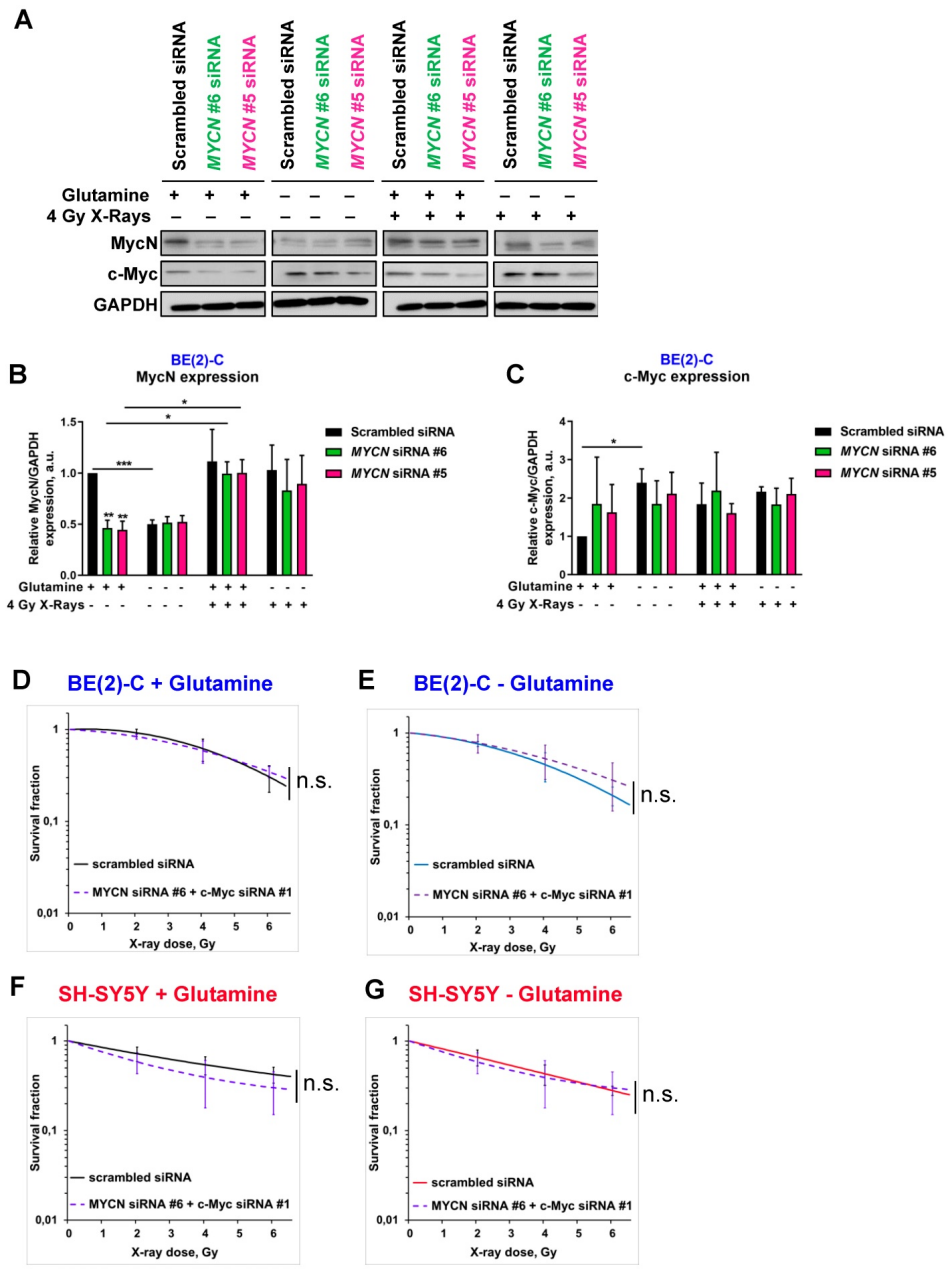
** Modulation of MYC member expression upon glutamine deprivation and irradiation.** (A) Representative western blot of MycN and c-Myc levels in BE(2)-C cells following *MYCN* downregulation upon glutamine deprivation and irradiation (4 Gy). GAPDH was used as a loading control. (B, C) Relative (B) MycN and (C) c-Myc protein expression analysed by densitometry in BE(2)-C cells following *MYCN* downregulation upon glutamine deprivation and irradiation (4 Gy) (n = 3; *p < 0.05; **p < 0.01; ***p < 0.001). (D, E) Radiobiological colony forming assay of BE(2)-C cells following *MYCN* and *c-MYC* downregulation (D) with glutamine or (E) upon 24 h glutamine deprivation (n ≥ 3; ± S.E.M; ns p > 0.05). (F, G) Radiobiological colony forming assay of SH-SY5Y cells following *MYCN* and *c-MYC* downregulation (F) with glutamine or (G) upon 24 h glutamine deprivation (n ≥ 3; ± S.E.M; ns p > 0.05).

To analyse the impact of glutamine deprivation in neuroblastoma cell radiosensitivity, we used radiobiological 3D colony forming analysis. Cells were cultured in glutamine-free media for 24 h and then irradiated with a single X-ray dose of 2, 4 or 6 Gy. Our results showed that 24 h glutamine deprivation led to a trend to radiosensitize SH-SY5Y and SK-N-AS cells, which is significant upon 48 h glutamine deprivation in SK-N-AS (Figures 3A and Figures S2A, B and C). In two *MYCN*-amplified cell lines, a significant increase in radioresistance was revealed upon 24 and 48 h glutamine deprivation in both BE(2)-C and Kelly cell lines (Figure 3B and Figures S2A, D and E). We also analysed the impact of glutamine deprivation on chemo-sensitivity in SH-SY5Y and BE(2)-C. A similar result was observed with chemoresistance to topotecan only in the *MYCN*-amplified BE(2)-C cell line (Figure S2F). As our previous data showed interplay between expression of Myc members upon glutamine deprivation, we first investigated the impact of MycN on cell radioresistance by modulating its expression. We performed the 3D colony forming assay after 24 h glutamine deprivation combined with different doses of X-rays in the two non-*MYCN*-amplified neuroblastoma cell lines, SH-SY5Y and SK-N-AS, stably overexpressing *MYCN* (Figure S1B). Our results showed a trend towards increased resistance to irradiation upon glutamine deprivation in both the cell lines expressing MycN (Figures S2G, H and I, comparison between colour lines). We then performed siRNA-mediated knockdown of *MYCN* in BE(2)-C cells and examined their clonogenic properties. No significant difference on the BE(2)-C radioresistant properties was observed after *MYCN* knockdown (Figures 3C and S3A and B). Our results however revealed that knockdown of *MYCN* restored the radiosensitivity of BE(2)-C cells upon glutamine deprivation (p<0.01; Figures 3D and S3A and C). As our results suggested a compensatory mechanism involving *MYCN* and *c-MYC* in *MYCN*-amplified neuroblastoma cells upon glutamine deprivation, we also performed the 3D colony-forming assay following *c-MYC* knockdown. In BE(2)-C cells, our data showed no effect on the radiosensitivity, independent of glutamine deprivation status (Figures 3E and F and S3D, E and F). In SH-SY5Y cells with high expression of c-Myc, its knockdown led to radiosensitization upon both glutamine-containing and glutamine-free conditions (Figures 3G and H and S3D, G and H). We next analysed Myc member protein levels by western blot following glutamine deprivation combined with irradiation in the BE(2)-C cells. Our data indicated that MycN and c-Myc protein levels were upregulated following irradiation (Figures 4A, B and C). Finally, a gene knockdown approach targeting both *MYCN* and *c-MYC* was used and the 3D colony-forming assay was performed. As shown in Figures S4A, B, C and D, *c-MYC* and *MYCN* mRNA levels were efficiently downregulated in both cell lines and independent of glutamine deprivation status. However, double knockdown of *MYC* members did not promote radiosensitization in either of the cell lines (Figures 4D, E, F, G and S4E). Collectively, these results highlight a complex interplay between *c-MYC* and *MYCN* that could regulate radioresistance properties upon glutamine deprivation in neuroblastoma cells.

### Glutamine deprivation modifies DNA repair gene pathway and redox balance in neuroblastoma cells

Our results showing a cell-line dependent effect on radioresistance properties upon glutamine deprivation highlighted the need for a better understanding of the underlying mechanism.

**Figure 5:**
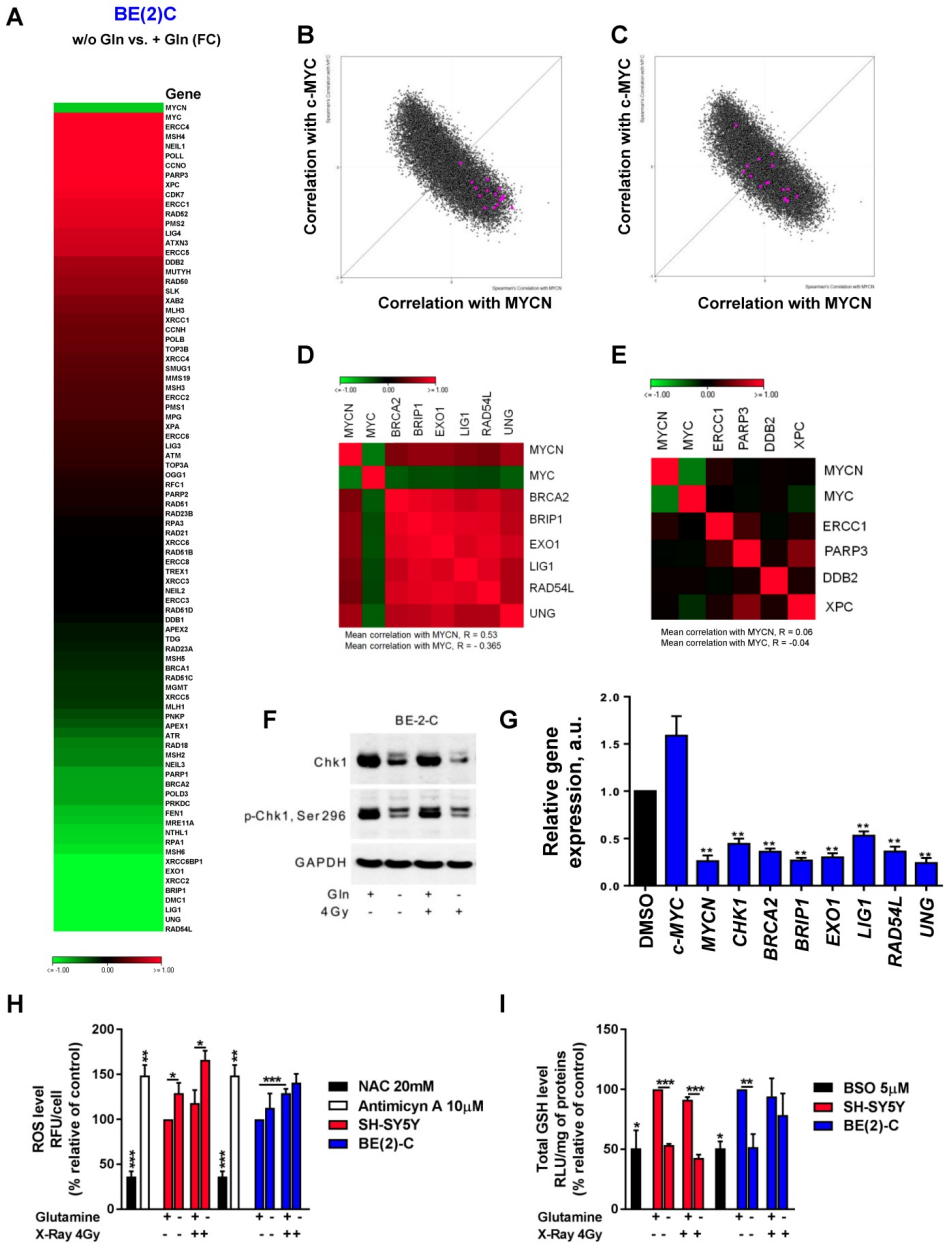
** Glutamine deprivation modifies DNA repair gene pathway and redox balance in neuroblastoma cells.** (A) Expression levels of 84 DNA repair genes analyzed using DNA RT² Profiler PCR Array Human DNA Repair in BE(2)-C cells upon 24 h of glutamine deprivation. Data are from two pooled experiments. (B, C) Correlation of mRNA expression for (B) 17 genes downregulated upon glutamine deprivation and (C) 16 genes upregulated upon glutamine starvation with *c-MYC* and *MYCN* genes in the neuroblastoma TARGET patient cohort. (D, E) Correlation map for (D) 6 clinically relevant genes, which were downregulated upon glutamine deprivation and (E) 4 clinically relevant genes, which were upregulated upon glutamine deprivation, with *c-MYC* and *MYCN*. (F) Representative western blot of total Chk1 and p-Chk1 levels in BE(2)-C cells upon glutamine deprivation and irradiation (4 Gy). GAPDH was used as a loading control. (G) Relative mRNA expression of *CHK1*, *MYCN*, *c-MYC* and 6 DNA repair genes upon glutamine deprivation in BE(2)-C cells. (H) Analysis of ROS levels in SH-SY5Y (red) and BE(2)-C (blue) cells upon 48 h glutamine deprivation and 4 Gy X-rays. NAC and antimycin A used as negative and positive controls. Data are reported as averages relative to control (n = 3; ± S.E.M; *p < 0.05; **p < 0.01; ***p < 0.001). (I) Analysis of total GSH levels in SH-SY5Y (red) and BE(2)-C (blue) cells upon 48 h glutamine deprivation and 4 Gy X-rays. BSO used as positive control. Data are reported as averages relative to control (n = 3; ± S.E.M; *p < 0.05; **p < 0.01; ***p < 0.001).

As radiation therapy induces cell death mediated through induction of DNA damage 30, we first evaluated the DNA repair gene pathway. We profiled expression of 84 genes involved in DNA repair signaling by qPCR array in BE(2)-C cells cultured in normal growth or glutamine-free media. Our data showed opposite gene expression levels between *MYCN* and *c-MYC* upon glutamine deprivation with a strong down-regulation of *MYCN* expression combined with an up-regulation of *c-MYC* expression, confirming our previous results (Figure 5A). Using a fold-difference cut-off of 1.5, we observed that glutamine deprivation repressed the expression of 17 key DNA repair genes and induced the expression of 16 genes (Table S2). To investigate the functional and clinical significance of these dysregulated genes, we extracted gene expression data of the paediatric neuroblastoma patients from TARGET study (2018, n=1,089 patients) and checked their co-expression with *MYC* members. Our analysis showed that the 17-downregulated genes upon glutamine deprivation were highly correlated with *MYCN* expression (Figure 5B), while no correlation was found with the 16-upregulated genes upon glutamine deprivation (Figure 5C). Generating a co-regulation map by selecting 6 out of the 17-dowregulated genes and 4 out of 16 upregulated genes based on their association with worse overall survival of neuroblastoma patients (Figures S5A and B), we found a high degree of inter-correlation that might reflect a functional relationship (Figure 5D), while no correlation between 4 genes upregulated upon glutamine deprivation was observed (Figure 5E). Our results suggest that upon glutamine deprivation, *MYCN* expression could modulate a specific group of DNA repair genes having a similar function in neuroblastoma. Furthermore, by deeper investigating the DNA pathway modulation upon glutamine deprivation in BE(2)-C cells, our data showed that total Chk1 and its phosphorylated form are downregulated (Figure 5F). We then used Prexasertib, a Chk1 inhibitor (Figure S6A) and analyzed the expression of the DNA repair gene signature (cluster of 6 genes) by qPCR. Our data demonstrated that inhibition of Chk1 correlated with expression changes of MycN and c-Myc upon glutamine deprivation (Figure 5G). Collectively, our results suggest that Chk1 can act as an upstream regulator of Myc member expression as well as DNA repair genes and be regulated by glutamine.

A number of different intrinsic and extrinsic adaptations might also confer high cellular radioresistance, including increased intracellular defense against irradiation-mediated ROS production 31. To determine whether glutamine deprivation also disrupts redox balance in neuroblastoma cells after irradiation, GSH and ROS levels were assessed. Glutamine deprivation resulted in a significant upregulation of ROS levels in SH-SY5Y cells, while only a small trend was shown in BE(2)-C cells (Figures 5H). Glutamine deprivation was associated with a significant decrease in GSH levels in both cell lines. Twenty-four hours post irradiation, our data showed an expected increase in ROS levels in both cell lines, while no effect was observed on the total GSH level (Figures 5I). Irradiation combined with glutamine deprivation led to a significant upregulation of ROS levels in the c-Myc expressing SH-SY5Y cells from 118 ± 15% in irradiated cells to 166 ± 10% (p > 0.05) in irradiated and glutamine-deprived cells. This was associated with a significant downregulation of GSH levels from 91 ± 2% in irradiated cells to 43 ± 3% (p < 0.001) in irradiated and glutamine-deprived cells. Conversely, no significant difference was observed in both ROS and GSH levels in the *MYCN*-amplified BE(2)-C cells when irradiation was combined with glutamine deprivation. Moreover, qPCR analysis of genes involved in antioxidant defence revealed that glutathione reductase (GSR) involved in recycling of oxidized glutathione (GSSG) to GSH was up-regulated in SH-SY5Y upon glutamine deprivation. At the same time expression of GCLC (Glutamate-Cysteine Ligase Catalytic Subunit) which is the first rate-limiting enzyme of glutathione synthesis as well as GGT1 (Glutathione hydrolase 1 proenzyme) responsible for degradation of GSSG were more highly upregulated in BE(2)-C cells (Figures S6B, C and D). These results suggest that BE(2)-C and SH-SY5Y can possess different dynamics of GSH turnover that is reflected on efficiency of the ROS scavenging upon glutamine deprivation. Collectively, the results of these experiments demonstrate that glutamine deprivation could lead to a selective radioresistance in neuroblastoma cells expressing MycN by increasing their ability to activate scavenging systems required for maintaining the intra-cellular redox balance.

### Glutamine deprivation modulates CSC properties of neuroblastoma cells

**Figure 6:**
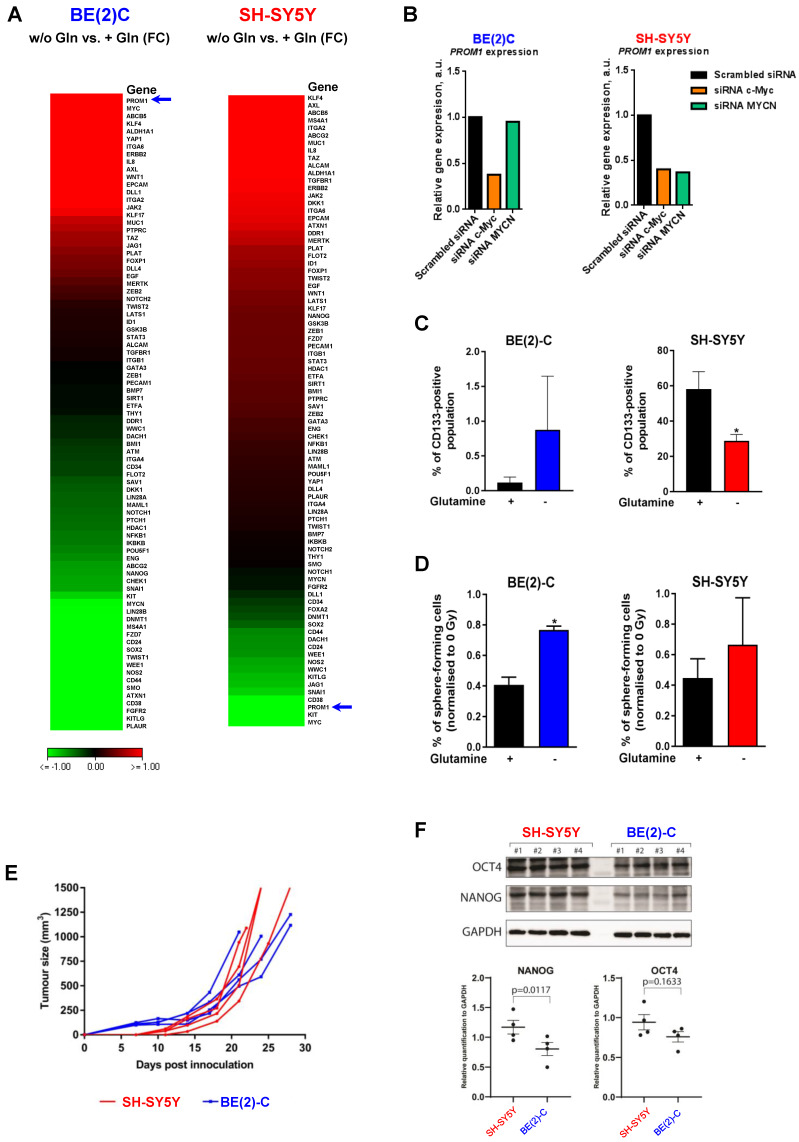
** Glutamine deprivation modulates CSC properties in neuroblastoma cells.** (A) Expression of 84 CSC-related genes analyzed by RT² Profiler PCR Array Human Cancer Stem Cells in BE(2)-C and SH-SY5Y cells upon 24 h of glutamine deprivation. Data are from two pooled experiments. (B) Relative expression level for *PROM1* following *c-MYC* or *MYCN* knockdown in BE(2)-C and SH-SY5Y cells. Data are from three pooled experiments. (C) Flow cytometry analysis of CD133-positive populations in BE(2)-C and SH-SY5Y cells upon glutamine deprivation (n ≥ 3; ± S.E.M; *p < 0.05). (D) Sphere forming assay of BE(2)-C and SH-SY5Y cells upon 24 h glutamine deprivation and 4 Gy X-rays. Data are reported as averages normalised to 0 Gy (n ≥ 3; ± S.E.M; *p < 0.05). (E) Tumor growth curves of xenografted SH-SY5Y and BE(2)-C cells (n = 4 for each cell line). (F) Representative western blot of OCT4 and NANOG levels in SH-SY5Y and BE(2)-C xenografted tumor samples. GAPDH was used as a loading control. Relative NANOG and OCT4 protein expression analysed by densitometry in SH-SY5Y and BE(2)-C xenografted tumor samples (n = 4 for each cell line).

**Figure 7:**
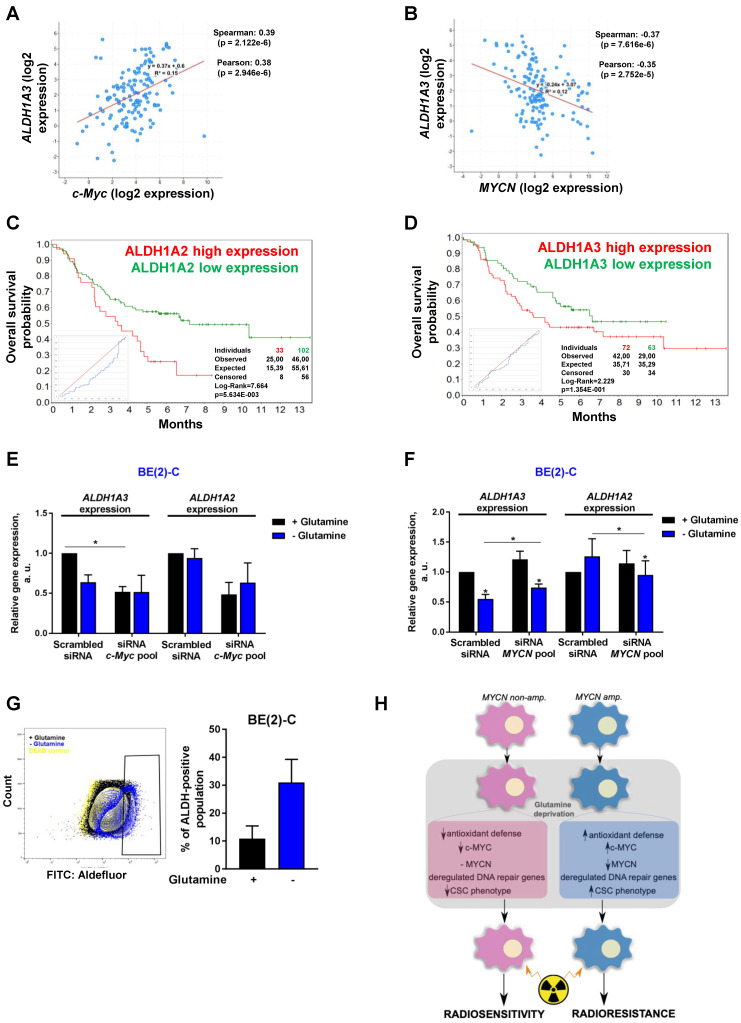
** Glutamine deprivation alters ALDH gene expression and activity in neuroblastoma cells.** (A, B) Correlation of mRNA expression for *ALDH1A3* with (A) *c-MYC* or (B) *MYCN*. R-values were determined using Pearson and Spearman correlation test. (C, D) Kaplan-Meier curves using the scan cut-off reporting patient overall survival depending on (C) *ALDH1A2* and (D) *ALDH1A3* expression levels. (E, F) Relative mRNA expression of *ALDH1A3* and *ALDH1A2* following (E) *c-MYC* or (F) *MYCN* downregulation upon 24 h glutamine deprivation in BE(2)-C cells (n = 3; ± S.E.M; *p < 0.05). (G) Flow cytometry analysis of ALDH positive population in BE(2)-C cells upon glutamine deprivation. Representative plots as illustration. (H) Schematic representation of the effect of glutamine deprivation on CSC and radioresistant properties in neuroblastoma cells depending on *Myc* member expression.

An increasing body of evidence demonstrates that CSCs are more radioresistant than the bulk of tumor cells 31. However, the dysregulation of CSC markers is still poorly investigated in neuroblastoma. By qPCR array, we profiled expression of 84 CSC-related genes in BE(2)-C and SH-SY5Y cells cultured in normal growth or glutamine-free media using the same fold-change cut-off of 1.5 as for DNA repair gene qPCR Array (Table S3). We observed that glutamine deprivation was able to differently modulate CSC-related genes in BE(2)-C and SH-SY5Y cells (Figure 6A). Mining the publically available neuroblastoma patient dataset TARGET, we showed that unlike DNA repair genes, CSC-related genes were preferentially correlating with expression of c-Myc (Figure S7A). A few marker-defined phenotypes of CSCs are described for neuroblastoma including CD133 encoded by the *PROM1* gene 13. qPCR array results showed that glutamine deprivation increased *PROM1* gene expression in BE(2)-C cells, while the opposite was observed in SH-SY5Y cells (Figure 6A, blue arrow). Analysis of the gene expression dataset from the TARGET study revealed that high expression of *PROM1* was significantly associated with a worst outcome for neuroblastoma patients (n = 68/247 Figure S7B). To further investigate the functional implication of *PROM1*, we first studied its expression following *MYC* member knockdown. Our results demonstrated that in SH-SY5Y knockdown of *c-MYC* or *MYCN* led to a downregulation of *PROM1* expression, while only *c-MYC* knockdown decreased its expression in BE(2)-C cells (Figures 6B and S7C and D). These data indicate that up-regulation of *c-MYC* upon glutamine deprivation could lead to an increase in CD133-positive BE(2)-C cells. We next evaluated CD133 expression by flow cytometry. Our results showed that both cell lines express a CD133-positive cell population, which is higher in SH-SY5Y than in BE(2)-C (Figure 6C). Glutamine deprivation led to an increase in the CD133+ population from 0.12 ± 0.05 to 0.88 ± 0.44% (p = 0.166) in BE(2)-C, while the CD133+ populations dropped down by 50.5 ± 3% (p < 0.05) in SH-SY5Y (Figure 6C). Moreover, our results demonstrated an up-regulation of ALDH1A3 and downregulation of CD44 proteins upon glutamine starvation in MYCN-amplified neuroblastoma cells, while a downregulation of both CSC markers was observed in SH-SY5Y cells (Figure S7E). To further analyse whether glutamine deprivation can modulate neuroblastoma CSC properties, we examined their spherogenic properties. Irradiation combined with glutamine deprivation significantly increased the sphere-forming population from 0.41 ± 0.05 to 0.77 ± 0.02% (p = 0.013) in BE(2)-C cells (Figure 6D). No significant difference in the sphere-forming population was observed in the c-Myc expressing SH-SY5Y cells (p = 0.4266; Figure 6D). Moreover *MYCN* gene silencing led to a small increase in the BE(2)-C sphere-forming cell population, which is significant for *MYCN* sequence 6 siRNA (Figure S7F). Finally, to appreciate the tumorigenicity of c-Myc and MycN cell lines, tumor growth rate was analysed *in vivo* using SH-SY5Y and BE(2)-C xenografted mouse models. Our results showed that tumorigenicity of both cell lines is similar (Figure 6E). Immunoblot analysis on mouse tumor samples revealed a higher expression in CSC markers in SH-SY5Y than in BE(2)-C, which is significant for the NANOG protein level expression (Figure 6F). Aldehyde dehydrogenase (ALDH) activity is also described as a CSC-marker in neuroblastoma 16. Our findings support a potential interplay between *MYCN* and *c-MYC* upon glutamine deprivation, and we initially sought to investigate the co-expression of CSC-related genes such as ALDH1A1 and ALDH1A3 with *MYC* member expression in the paediatric neuroblastoma patient data from the TARGET study. Expression of both CSC-related genes had a modest positive correlation with *c-MYC* expression (Figures 7A and S8A), whereas a modest negative correlation was revealed with *MYCN* expression (Figures 7B and S8B). This data suggests the existence of a distinct pattern of co-expression of CSC-related genes with *MYC* members in patient samples. We next focused our analyses on the expression of ALDH genes related to neuroblastoma CSCs, including ALDH1A2 and ALDH1A3. Analysis of the gene expression dataset from the TARGET study revealed that high expression of both *ALDH1A2* and *ALDH1A3* was associated with a worst outcome for neuroblastoma patients (n= 33/135 and 72/135 for *ALDH1A2* and *ALDH1A3* respectively; Figures 7C and D). To investigate the impact of glutamine deprivation on *ALDH* gene expression, we used qPCR assay upon glutamine deprivation alone or in combination with *c-MYC* or *MYCN* knockdown in BE(2)-C cells. Knockdown of *c-MYC* in BE(2)-C cells resulted in downregulation of *ALDH1A3* and *ALDH1A2* mRNA expression independently of glutamine deprivation status (Figure 7E). In contrast, *MYCN* knockdown led to a slight increase in *ALDH1A3* and *ALDH1A2* expression (Figure 7F). Moreover, we assessed the presence of ALDH-positive neuroblastoma cells by flow cytometry. Our results showed that ALDH-positive cells increased from 10.9 ± 4.6 to 31 ± 8.4% (p = 0.076; Figure 7G) upon glutamine deprivation. The same analysis was performed in SH-SY5Y cells. No significant difference was observed in the ALDH-positive population in SH-SY5Y cells upon glutamine starvation (p = 0.356; Figure S8C). Taken together, our results suggest that glutamine deprivation can specifically modulate expression of *MYC* members and CSC phenotype in neuroblastoma cells and could correspond to the changes in the radioresistant properties (Figure 7H).

## Discussion

The dependence of cancer cells on glutamine metabolism has made it an attractive therapeutic target. In this study, we focused on high-risk neuroblastoma harbouring high-level expression of Myc members. As radiotherapy is one of the main curative options, we studied the therapeutic potential of combining irradiation with glutamine deprivation. Our results demonstrate interplay between Myc members in regulation of neuroblastoma CSC phenotype and radioresistance properties upon glutamine deprivation.

The prominent role of Myc member expression in neuroblastoma implies that Myc proteins are likely to be an important therapeutic target, presenting a unique opportunity for a targeted strategy in neuroblastoma 3, 5, 32. Nevertheless, to date, no effective treatment demonstrating convincing evidence of Myc inhibition has yet been translated into the clinic. Therapeutic strategies to indirectly target Myc proteins have emerged, including blocking Myc-dependent transcription or targeting regulators of *MYC* mRNA and protein stability 5, 33. Recently, interest in the therapeutic targeting of glutamine metabolism in Myc-driven cancers has emerged, leading to the development of a number of classes of glutamine metabolism inhibitors 21. Herein, our results showed that glutamine deprivation were associated with a decrease in cell proliferation in all cell lines tested, while differences in cell responses were observed for cell viability. Previous studies have already reported that sensitivity of cancer cells to glutamine deprivation is context dependent, such that glutamine deprivation in human lung 34 or breast cancer 35 cell lines resulted in a wide spectrum of responses. This result might be explained by genomic differences such as mutations that vary among different cancer types and cell lines.

Our data showed that glutamine deprivation led to a downregulation of MycN and c-Myc expression at both mRNA and protein levels in neuroblastoma cells harbouring *MYCN* amplification or high c-Myc expression respectively. Previous studies 36-38 have shown that c-Myc and MycN have incomplete redundancy and these results are furthermore supported by our analysis mining the patients' data showing an inverse correlation between *c-MYC* and *MYCN* expression as well as gene signatures. In this study, we observed a specific regulatory interplay between expression of Myc members in the *MYCN*-amplified neuroblastoma cells. This result could explain a high therapeutic resistance observed in *MYCN*-amplified neuroblastoma patients as Myc members are well known to be implicated in chemoresistance and radioresistance 5. Further studies will therefore be necessary to reveal possible upstream targets regulating Myc member impact on the radioresistance mechanism. One possible target could be Max interactor (Mxi1) known to promote Myc activation and radioresistance in lung cancer cells 39.

Because monotherapies are not always able to cure advanced cancer, we next investigated the effect of glutamine deprivation in combination with radiation therapy, which is one of the mainstays of treatment for high-risk neuroblastoma 7. The risk of relapse presents a significant clinical challenge. Previous studies have demonstrated that glutamine metabolism plays an essential role in radioresistance mechanisms in different types of cancers including breast, prostate as well as colorectal cancers 40-42. In lung cancer several studies have suggested the potential therapeutic benefit of glutamine inhibition to increase radiation sensitivity 43, 44. Our results revealed that glutamine deprivation synergizes with radiation therapy to radiosensitize neuroblastoma cells harbouring c-Myc expression. However, we demonstrated for the first time that combined glutamine deprivation with radiation therapy in *MYCN*-amplified neuroblastoma led to an increase in radioresistant cells that is associated with upregulation of c-Myc expression in these cells.

Two of the major effects of radiotherapy are to induce cellular damage directly *via* lethal damage to the DNA and indirectly *via* the production of ROS. Here, a redox balance disruption was observed in the radiosensitive SH-SY5Y cells, while the *MYCN*-amplified BE(2)-C cells were able to maintain GSH and ROS levels upon glutamine deprivation when combined with radiation therapy. It has become clear that increased intracellular defense against irradiation-mediated ROS production confers high cellular radioresistance 45. In a previous study, Yogev *et al.* have revealed that the principal acquired mechanism of radioresistance in neuroblastoma was a metabolic adaptation through increased production of GSH in response to chronic oxidative stress 46. In our study, as Myc member expression increased following radiotherapy in BE(2)-C cells, Myc-driven upregulation of glutamine pathway genes may have led to metabolic adaptation leading to cell radioresistance. Moreover, our data showed that glutamine deprivation can modulate the DNA repair gene signature, and *c-Myc* and *MYCN* expression is associated with expression of different DNA repair regulatory genes. As tumor cells with highly efficient DNA repair are radioresistant 30, this opposite gene signature pattern depending on Myc member expression could explain the different radioresistance properties upon glutamine deprivation observed in *MYCN*-amplified and c-Myc expressed neuroblastoma cells. A group of specific genes, which is highly correlated to *MYCN* expression and having a co-regulation pattern has been found significantly dysregulated upon glutamine deprivation. In particular, our results highlighted that Chk1 could be an upstream regulator of Myc member expression upon glutamine deprivation and might represent a key protein.

A growing body of evidence is indicating that radiotherapy has the potential to kill highly proliferative bulk tumor cells and reduce tumor burden, but a small number of malignant cells with CSC features might survive and regrow the tumor 8-10. Recently, a combined approach of targeting CSC metabolism in conjunction with the use of chemotherapy or radiotherapy has emerged as a promising strategy to overcome cancer cell resistance 17. Here, for the first time, we demonstrated that glutamine deprivation led to an enhanced expression of the CD133 CSC marker as well as an increase in the spherogenic potential specifically in the *MYCN*-amplified cell line. Multiple studies have shown the direct involvement of Myc members in regulation of CSC populations and radioresistance in different tumor entities 47-49. As demonstrated by the up-regulation of c-Myc levels upon glutamine deprivation or *MYCN* gene knockdown, these results might explain a regulatory interplay between expression of Myc members to regulate CSC properties. However, our results showed that glutamine deprivation combined with double Myc member knockdown did not significantly alter the cell radiosensitivity, while *MYCN* downregulation in BE(2)-C or *c-MYC* downregulation in SH-SY5Y cells led to radiosensitisation of the cells. This data suggests the existence of other molecular mechanisms (*e.g.* metabolism-related) controlling the complex interplay between c-Myc and MycN expression in neuroblastoma cells, as well as the importance of Myc family-related pathways for sustaining neuroblastoma cell survival. Further studies focusing on genes regulating glutamine pathway could be necessary as few genes are already known to promote the stemness of cancer cells 50.

The catalytic activity of ALDH can be used as a marker to identify CSCs in the different tumor entities including neuroblastoma. Previous studies including our findings have already demonstrated that its activity is correlated with the prognosis of certain groups of cancer patients, including prostate or breast cancers 51-53 and is upregulated in the radioresistant CSC populations 28. The level of ALDH activity depends on the expression levels and posttranslational modifications of several ALDH1 proteins including ALDH1A1, ALDH1A3 and ALDH1A2. Recent studies identified ALDH1A2 and ALDH1A3 genes as markers of neuroblastoma CSC 16. Our data revealed that ALDH1A1 and ALDH1A3 mRNA expression positively correlates with *c-MYC* gene expression in the neuroblastoma patient samples, and siRNA-mediated *c-MYC* knockdown inhibited expression of ALDH1A2 and ALDH1A3 genes in the *MYCN*-amplified neuroblastoma cells. Moreover, by mining publically available data, our bioinformatics analysis indicated that neuroblastoma patients with low *ALDH* gene expression would have a better outcome than neuroblastoma patients with high *ALDH* gene expression. Future studies will need to address the exact underlying mechanism to better understand the radioresistance processes involving Myc members and CSC-markers.

## Conclusion

In conclusion, our findings have identified a potential interplay between Myc members in response to glutamine deprivation combined with irradiation. These results add to the body of literature about the complexity of Myc member targeting as well as the complexity of glutamine sensing and cellular responses that are likely cell type specific and context dependent. Further studies are required to better understand the radioresistance mechanisms in neuroblastoma in order to select patients who are likely to benefit from glutamine metabolism treatment.

## Abbreviations

BSO: buthionine sulfoximine
CSCs: cancer stem cells
GSH: glutathione
NAC: N-acetyl cystein
ROS: reactive oxygen species
TCA: tricarboxylic acid cycle
