# Supplementary material for: Interplay between MycN and c-Myc regulates radioresistance and cancer stem cell phenotype in neuroblastoma upon glutamine deprivation
Source: Theranostics. 2020 May 16;10(14):6411–29. doi: 10.7150/thno.42602 (PMC7255021; doi:10.7150/thno.42602)
Supplement: Supplementary file 1 — Supplementary figures and tables. [file thnov10p6411s1.pdf]

SUPPLEMENTARY DATA

Supplementary Figure 1: (A) Growth inhibition assay performed on two neuroblastoma cell lines using Alamar Blue after 72 h incubation with a range of concentrations of CB-839 ($n \geq 3$; ± S.E.M). (B) Western blot analysis of ectopic MycN expression in protein extracts from SHEP/TET21N inducible cell line, SH-SY5Y and SK-N-AS cell lines transfected with empty control pCDH vector or pMYCN vector. GAPDH was used as a loading control. (C, E, G) Cell number and (D, F, H) cell viability performed using Trypan Blue Dye exclusion assay on neuroblastoma cells with ectopic MycN expression cultured with or without glutamine from 24 to 72 h. Data are reported as averages ($n \geq 3$; ± S.E.M; ns $p > 0.05$; *$p < 0.05$; **$p < 0.01$).

Supplementary Figure 2: (A) Plating efficiency of neuroblastoma cells upon 24 h glutamine deprivation ($n \geq 3$; ± S.E.M). (B) Radiobiological colony forming assay of SH-SY5Y cells upon 48 h glutamine deprivation ($n \geq 3$; ± S.E.M). (C) Radiobiological colony forming assay of SK-N-AS cells upon 24 or 48 h glutamine deprivation ($n \geq 3$; ± S.E.M; ***$p < 0.001$). (D) Radiobiological colony forming assay of BE(2)-C cells upon 48 h glutamine deprivation ($n \geq 3$; ± S.E.M; **$p < 0.01$). (E) Radiobiological colony forming assay of Kelly cells upon 24 or 48 h glutamine deprivation ($n \geq 3$; ± S.E.M; ***$p < 0.001$). (F) Growth inhibition assay performed on two neuroblastoma cell lines using Alamar Blue after 72 h incubation with a range of concentrations of topotecan following 48 h glutamine deprivation ($n \geq 3$; ± S.E.M). (G) Plating efficiency of SK-N-AS and SH-SY5Y cells with ectopic MycN expression upon 24 h glutamine deprivation ($n \geq 3$; ± S.E.M). (H, I) Radiobiological colony forming assay of (H) SH-SY5Y cells and (I) SK-N-AS cells with ectopic MycN expression upon 24 h glutamine deprivation ($n \geq 3$; ± S.E.M; ns $p > 0.05$).

Supplementary Figure 3: (A) Plating efficiency of BE(2)-C cells upon 24 h glutamine deprivation and *MYCN* downregulation ($n \geq 3$; ± S.E.M). (B, C) Radiobiological colony forming assay of BE(2)-C cells following *MYCN* downregulation (B) with glutamine or (C) upon 24 h glutamine deprivation ($n \geq 3$; ± S.E.M; ns $p > 0.05$; **$p < 0.01$). (D) Plating efficiency of BE(2)-C and SH-SY5Y cells upon 24 h glutamine deprivation and *c-MYC* downregulation ($n \geq 3$; ± S.E.M). (E, F) Radiobiological colony forming assay of BE(2)-C cells following *c-MYC* downregulation (E) with glutamine or (F) upon 24 h glutamine deprivation ($n \geq 3$; ± S.E.M; ns $p > 0.05$). (G, H) Radiobiological colony forming assay of SH-SY5Y cells following *c-MYC* downregulation (G) with glutamine or (H) upon 24 h glutamine deprivation ($n \geq 3$; ± S.E.M; ns $p > 0.05$).

Supplementary Figure 4: (A, B) Relative mRNA expression of (A) *MYCN* and (B) *c-MYC* following *MYCN* and *c-MYC* downregulation upon glutamine deprivation in BE(2)-C cells. (C, D) Relative mRNA expression of (C) *c-MYC* and (D) *MYCN* following *MYCN* and *c-MYC* downregulation upon glutamine deprivation in SH-SY5Y cells ($n = 3$; *$p < 0.05$; **$p < 0.01$). (E) Plating efficiency of BE(2)-C and SH-SY5Y cells upon 24 h glutamine deprivation and *MYCN* and *c-MYC* downregulation ($n \geq 3$; ± S.E.M).

Supplementary Figure 5: (A, B) Correlation of mRNA expression for (A) 6 genes downregulated and (B) 4 genes upregulated upon glutamine deprivation with overall survival of neuroblastoma patients from the TARGET study. Median cut-off used; green is low gene expression level ($n = 124$) and red is high gene expression level ($n = 125$).

Supplementary Figure 6: (A) Representative western blot of total Chk1 and p-Chk1 levels

in BE(2)-C cells following 48 h prexasertib treatment. GAPDH was used as a loading control. (B, C) Relative mRNA expression of 8 antioxidant genes upon glutamine deprivation and irradiation in (B) BE(2)-C and (C) SH-SY5Y cells (n = 3; *p < 0.05; **p < 0.01. (D) Relative gene expression changes of 3 antioxidant genes between BE(2)-C and SH-SY5Y cells (*p < 0.05; **p < 0.01).

Supplementary Figure 7: (A) Correlation of mRNA expression for 84 CSC-related genes with *c-Myc* and *MYCN* in neuroblastoma TARGET patient cohort. (B) Correlation of *PROM1* mRNA expression with overall survival of neuroblastoma patients from the TARGET study. Scan cut-off; green is low gene expression level (n = 179) and red is high gene expression level (n = 68). (C) Validation of *c-MYC* knockdown in BE(2)-C and SH-SY5Y cells (n ≥ 3; ± S.D; **p < 0.01; ***p < 0.001). (D) Validation of *MYCN* knockdown in BE(2)-C and SH-SY5Y cells (n ≥ 3; ± S.D; ***p < 0.001). (E) Representative western blot of ALDH1A3 and CD44 levels in SH-SY5Y, BE(2)-C and Kelly cells upon glutamine deprivation. GAPDH was used as a loading control. (F) Sphere forming assay of BE(2)-C cells following *MYCN* downregulation upon 24 h glutamine deprivation and 4 Gy X-rays. Data are reported as averages normalised to 0 Gy (n ≥ 3; ± S.E.M; ns p > 0.05; **p < 0.01).

Supplementary Figure 8: (A, B) Correlation of mRNA expression for *ALDH1A1* with (A) *c-MYC* or (B) *MYCN*. R-values were determined using Pearson and Spearman correlation test. (C) Flow cytometry analysis of ALDH positive population in SH-SY5Y cells upon glutamine deprivation. (n ≥ 3; ± S.E.M).

Supplementary Table 1: Sequences of siRNAs and oligonucleotides and list of antibodies

Supplementary Table 2: DNA repair genes deregulated (> 1.5-fold change) in BE(2)-C upon glutamine deprivation for 24 h. A pooled analysis was performed with two independent biological repeats. Gene expression data was normalized to expression of five reference genes (*ACTB, GAPDH, B2M, RPLP0,* and *HPRT1*).

Supplementary Table 3: CSC-related genes deregulated (> 1.5-fold change) in BE(2)-C and SH-SY5Y cells upon glutamine deprivation for 24 h. A pooled analysis was performed with two independent biological repeats. Gene expression data was normalized to expression of five reference genes (*ACTB, GAPDH, B2M, RPLP0,* and *HPRT1*).

Table S1. Nucleotide sequences of siRNAs, primers used for qPCR and antibodies used for Western blotting and flow cytometry

**siRNA**	**Nucleotide sequence**
c-Myc #1	5’-GGAACGAGCUAAAACGGAG-3’
c-Myc #2	5’-GGAGGAGAACUUCUACCAG-3’
MycN #5	5’-CGGACGAAGAUGACUUCUATT -3’
MycN #6	5’-UGCCGGAGUUGGUAAAGAATT-3’
MycN #3	5’-GGCCACUGAGUAUGUCCACTC-3’
c-Myc SMARTpool	5’-ACGGAACUCUUGUGCGUAA-3’;<br>5’-GAACACACAACGUCUUGGA-3’;<br>5’-AACGUUAGCUUCACCAACA-3’;<br>5’-CGAUGUUGUUUCUGUGGAA-3’
**Oligonucleotide primers**	**Nucleotide sequences**
c-Myc	Forward: 5’-CTCCGTCCTCGGATTCTCTGC-3’<br>Reverse: 5’-CTCCAGCAGAAGGTGATCCAG-3’
MycN	Forward: 5’-CCTGAGCGATTCAGATGATG-3’<br>Reverse: 5’-CTTGGGACGCACAGTGATGG-3’
ACTB	Forward: 5’-ATGGAGTCCTGTGGCATCCA-3’<br>Reverse: 5’-AGTACTTGCGCTCAGGAGGA-3’
ALDH1A2	Forward: 5’-TTGCAGGGCGTCATCAAAAC-3’<br>Reverse: 5’-ACACTCCAATGGGTTCATGTC-3’
ALDH1A3	Forward: 5’-TCTCGACAAAGCCCTGAAGT-3’<br>Reverse: 5’-TATTCGGCCAAAGCGTATTC-3’
CD133/PROM1	Forward: 5’-GGATTATTCTATGCTGTGTCCTG-3’<br>Reverse: 5’-TGCCACAAAACCATAGAAGAT-3’
EXO1	Forward: 5’-AACTAGCCAAAGGTGAACCTACT-3’<br>Reverse: 5’-TGTGATATTGATAGACCGGGTGA-3’
BRIP1	Forward: 5’-CTTACCCGTCACAGCTTGCTA-3’<br>Reverse: 5’-CACTAAGAGATTGTTGCCATGCT-3’
LIG1	Forward: 5’-GCCCTGCTAAAGGCCAGAAG-3’<br>Reverse: 5’-CATGGGAGAGGTGTCAGAGAG-3’
UNG	Forward: 5’-CCCACACCAAGTCTTCACC-3’<br>Reverse: 5’-TTGAACACTAAAGCAGAGCC-3’
RAD54L	Forward: 5’-TTGAGTCAGCTAACCAATCAACC-3’<br>Reverse: 5’-GGAGGCTCATACAGAACCAAGG-3’
CHK1	Forward: 5’-CCAGATGCTCAGAGATTCTTCCA-3’<br>Reverse: 5’-TGTTCAACAAACGCTCACGATTA-3’
BRCA2	Forward: 5’-CCACCACCACACAGAATTCT-3‘<br>Reverse: 5‘-ATGCAATAAACCTGAATCAGC-3‘
GCLC	Forward: 5’-CAGGAAGGCATTGATCATCTC-3‘<br>Reverse: 5‘-CCATGGGTCGAAATTCTACTC-3‘
GSR	Forward: 5’-ACACTGGGACTCACGGAAG-3’<br>Reverse: 5’-CTGCATATCGATCCCAACCAC-3’
SOD2	Forward: 5’-GGTTTTGGGGTATCTGGGCTCCA-3’<br>Reverse: 5’-CTTGGCCAACGCCTCCTGGTA-3’
CAT	Forward: 5’-ACGTCTGTCTGAGAACATTG-3’<br>Reverse: 5’-CTTAGGGTTCTCAGCATTGTA-3’

PRDX6	Forward: 5’-GACTGCCCTTTCAATAGACAG-3’<br>Reverse: 5’-CCCTTTTCATCCTTCTCTGC-3’
GCLM	Forward: 5’-CGAGGAGCTTCATGATTGTATCC-3‘<br>Reverse: 5‘-CCATGTCAACTGCACTTCTAG-3‘
GGT1	Forward: 5’-GATGCCAACCAGTGCTCGAAG-3’<br>Reverse: 5’-GACCTCAGCTTTTCGTGTGG-3’
GPX1	Forward: 5’-GAGAACCCCAAGAACGAAGAG-3’<br>Reverse: 5’-GCTTGGGGTCGGTCATAAG-3’
**Antibodies / application**	**Manufacturer ID**
ALDH1A3 / Western blotting	Sigma-Aldrich, HPA046271, #ABN427
CD44 / Western blotting	Santa Cruz Biotechnology, HCAM (F-4), #sc-9960
Chk1 / Western blotting	Cell Signaling Technology, #2360 S
pChk1 S296 / Western blotting	Cell Signaling Technology, #2349 P
GAPDH / Western blotting	Abcam, #ab8245
MycN / Western blotting	SantaCruz, #sc-53993
c-Myc / Western blotting	**Cell signaling, #9402S**
PARP / Western blotting	Cell Signaling 46D11, #9532S
cleaved-PARP / Western blotting	Cell Signaling D64E10, #5625S
NANOG / Western blotting	Cell Signaling, #3580
Oct4 / Western blotting	Cell Signaling, #2750
CD133-PE / flow cytometry	Miltenyi Biotec, clone 293C3, #5181106100
CD117-PE / flow cytometry	Miltenyi Biotec, clone A3C6E2, #5181106146
IgG2b-PE / flow cytometry	Miltenyi Biotec, #5181106018
IgG-PE / flow cytometry	Miltenyi Biotec, #5181106103

Supplementary Table 2

**BE(2)C cells**	
Gene Symbol	FC normalized, -Glutamine vs. +Glutamine
ERCC4	3.21513
MSH4	2.89396
NEIL1	2.47493
POLL	2.36075
CCNO	2.22598
PARP3	2.0607
XPC	1.93042
CDK7	1.89513
ERCC1	1.82668
RAD52	1.81086
PMS2	1.79522
LIG4	1.72232
ATXN3	1.70599
ERCC5	1.70198
DDB2	1.54392
MUTYH	1.52226
BRCA2	0.64222
PARP1	0.64061
POLD3	0.63606
PRKDC	0.63483
FEN1	0.59619
MRE11A	0.58675
NTHL1	0.56361
RPA1	0.55873
MSH6	0.53933
XRCC6BP1	0.50634
EXO1	0.49386
XRCC2	0.44326
BRIP1	0.43229
DMC1	0.42506
LIG1	0.40338
UNG	0.34595
RAD54L	0.28717

Supplementary Table 3

BE(2)C cells		SH-SY5Y cells	
Gene Symbol	FC normalized, - Gln vs. + Gln	Gene Symbol	FC normalized, - Gln vs. + Gln
PROM1	17.28438	KLF4	5.834816
MYC	12.5978	AXL	3.85999
ABCB5	11.43151	ABCB5	3.597756
KLF4	7.116205	MS4A1	3.078209
ALDH1A1	5.575478	ITGA2	2.294654
YAP1	4.201265	ABCG2	2.225608
ITGA6	3.680077	MUC1	2.173072
ERBB2	3.360114	IL8	2.143426
IL8	2.844567	TAZ	2.051051
AXL	2.463906	ALCAM	2.039392
WNT1	2.428304	ALDH1A1	2.016397
EPCAM	2.407676	TGFBR1	1.979288
DLL1	2.181817	ERBB2	1.977277
ITGA2	2.111061	JAK2	1.928972
JAK2	2.056308	DKK1	1.916037
KLF17	1.928385	ITGA6	1.89101
MUC1	1.710341	EPCAM	1.865814
PTPRC	1.636532	ATXN1	1.814993
TAZ	1.540456	DDR1	1.703605
JAG1	1.531764	MERTK	1.667524
ABCG2	0.652738	PLAT	1.541546
NANOG	0.647073	FLOT2	1.535867
CHEK1	0.637661	WEE1	0.666291
SNAI1	0.634334	NOS2	0.635919
KIT	0.564193	WWC1	0.62265
MYCN	0.50647	KITLG	0.601951
LIN28B	0.501862	JAG1	0.593522
DNMT1	0.486316	SNAI1	0.566271
MS4A1	0.460024	CD38	0.372905
FZD7	0.458467	PROM1	0.2781
CD24	0.452182	KIT	0.244557
SOX2	0.414181	MYC	0.140218
TWIST1	0.411908		
WEE1	0.379966		
NOS2	0.376196		
CD44	0.35879		
SMO	0.357417		
ATXN1	0.284906		
CD38	0.278292		
FGFR2	0.220686		
KITLG	0.151845		
PLAUR	0.148664		

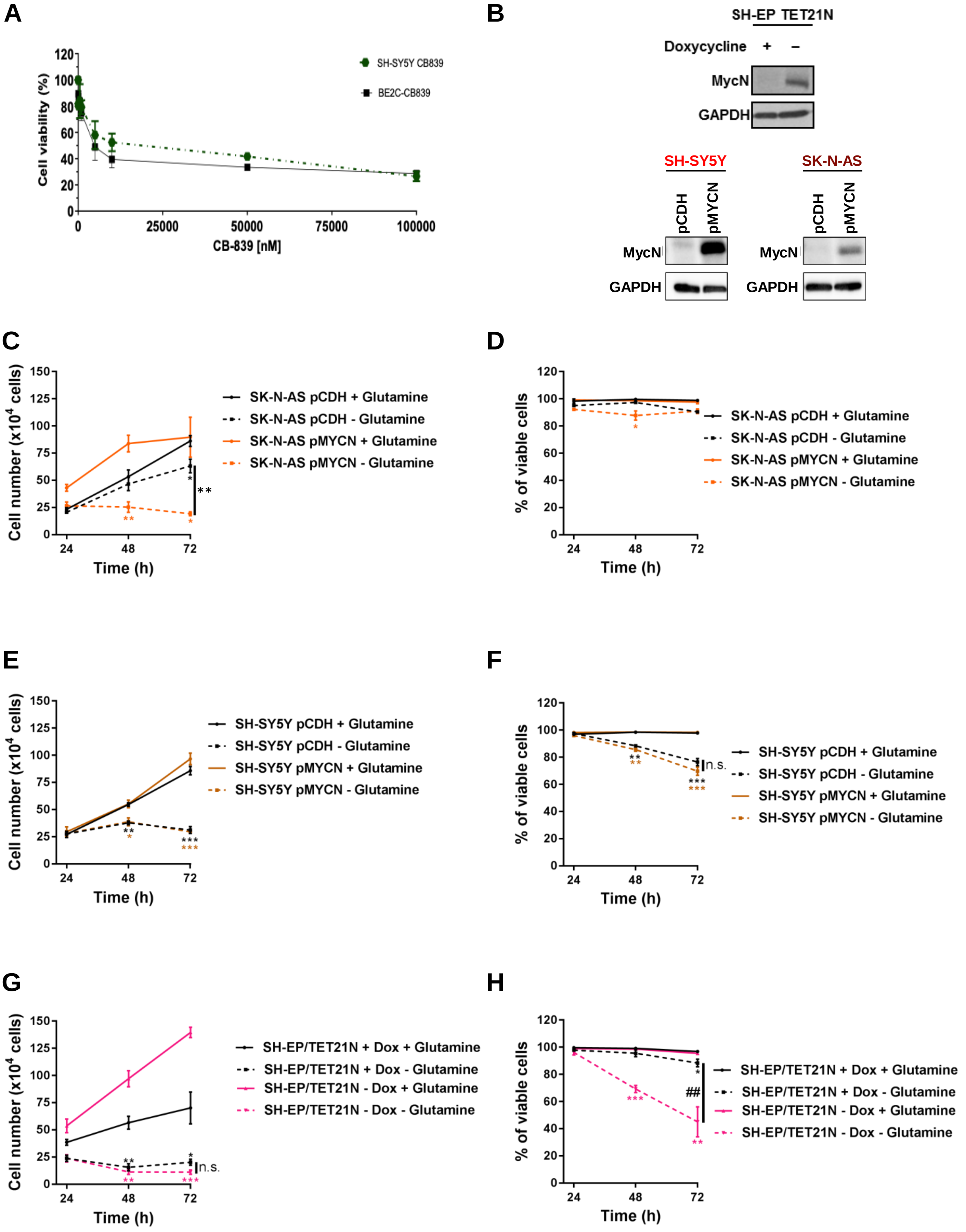

Supplementary Figure 1

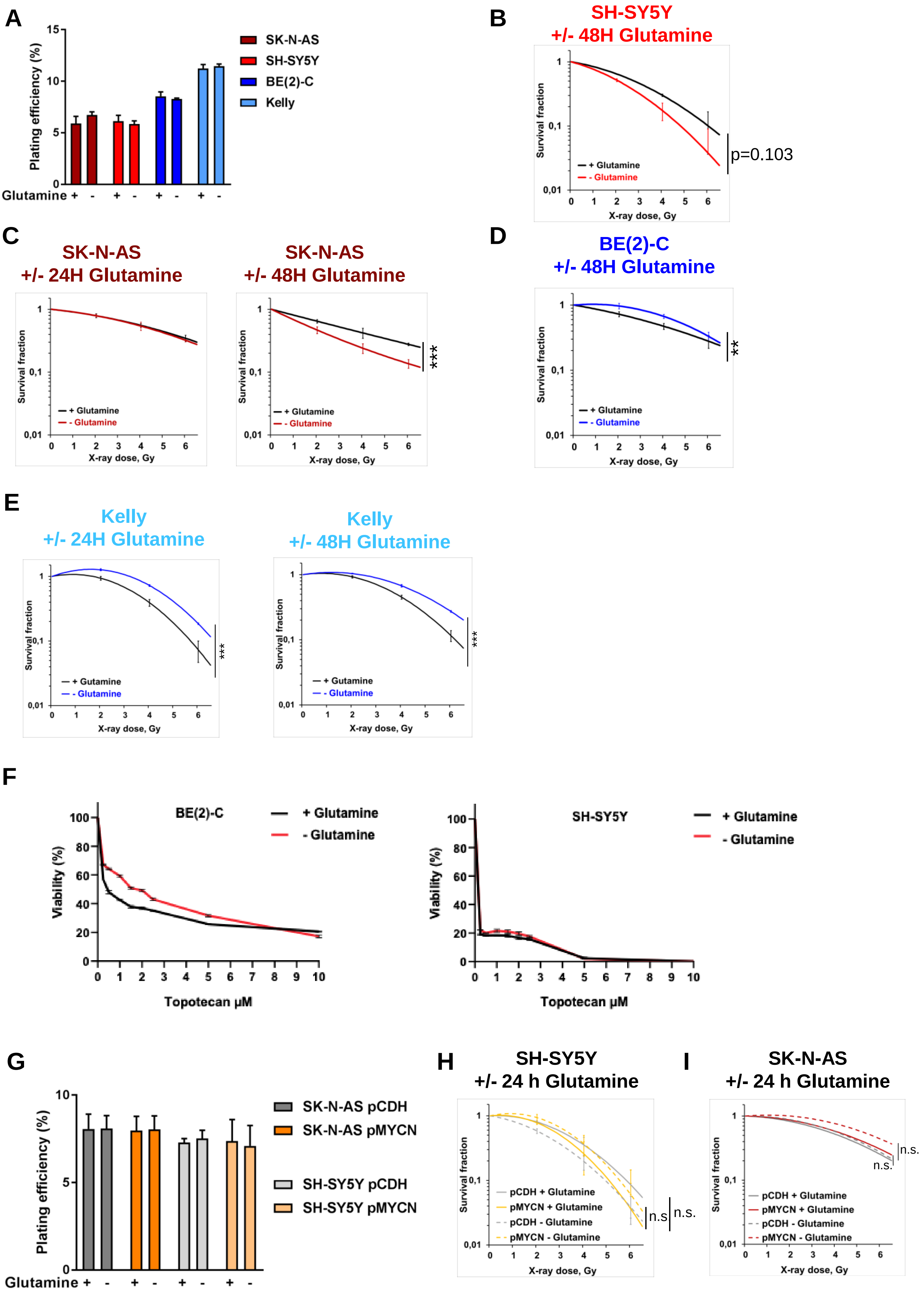

Supplementary Figure 2

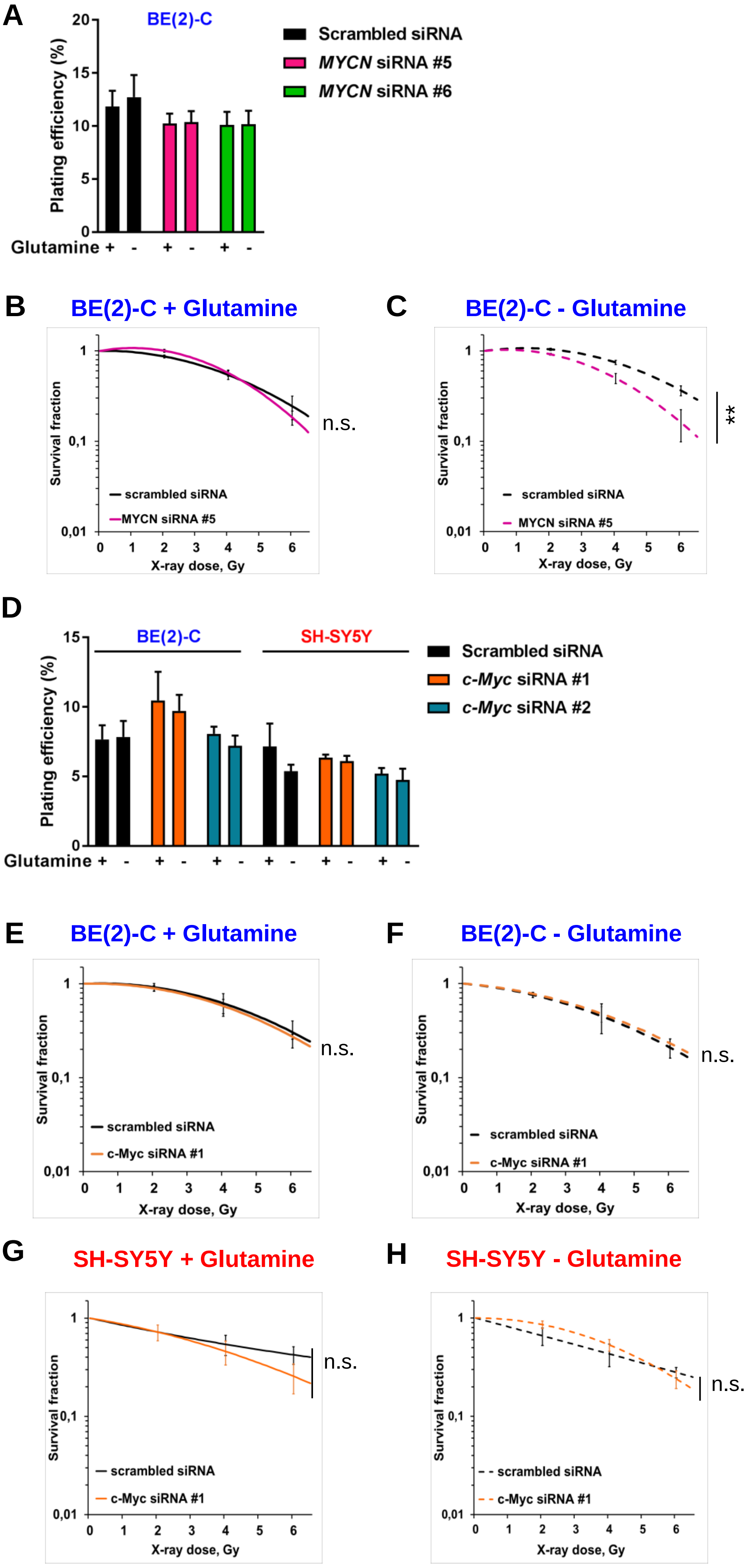

Supplementary Figure 3

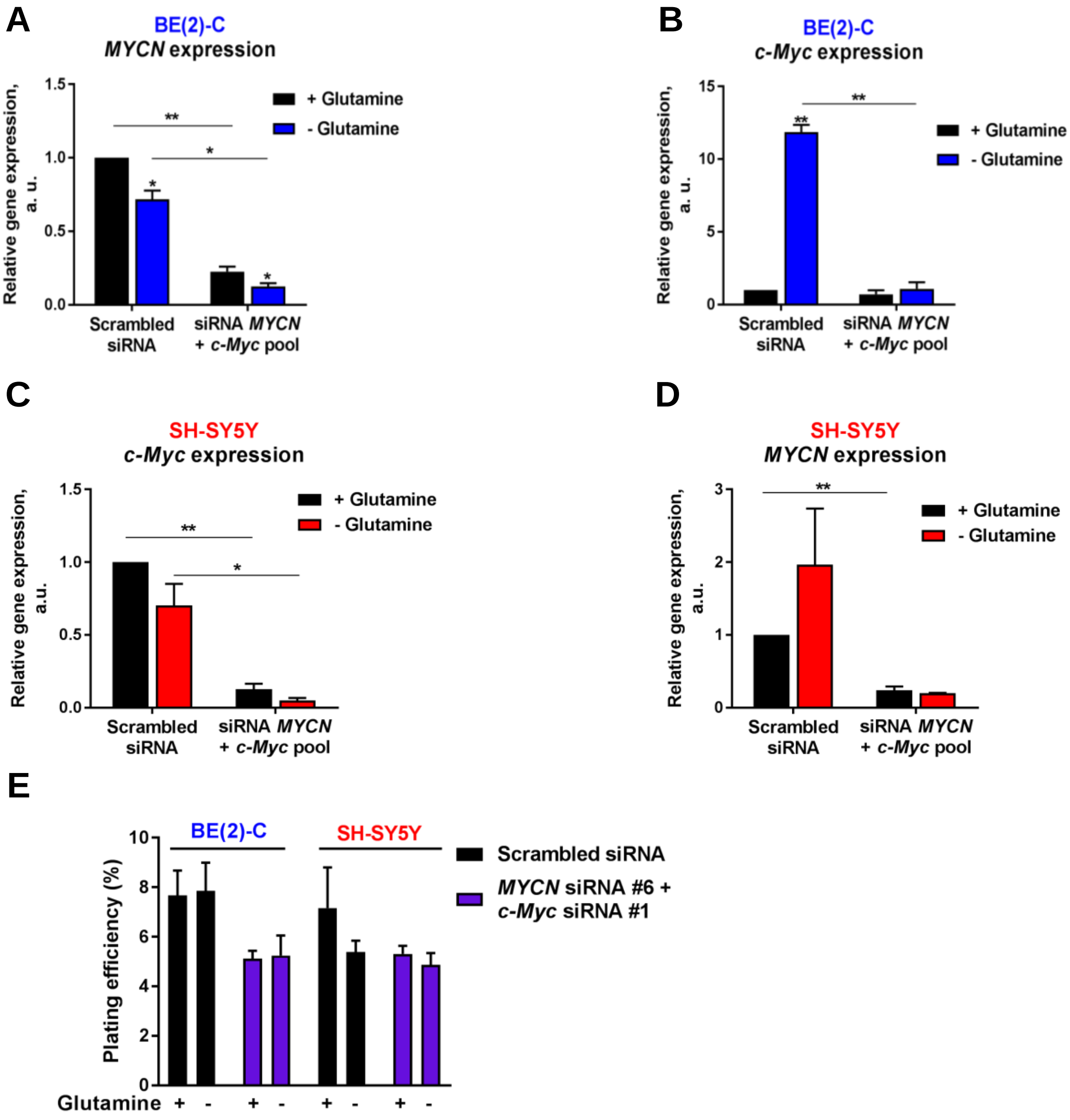

Supplementary Figure 4

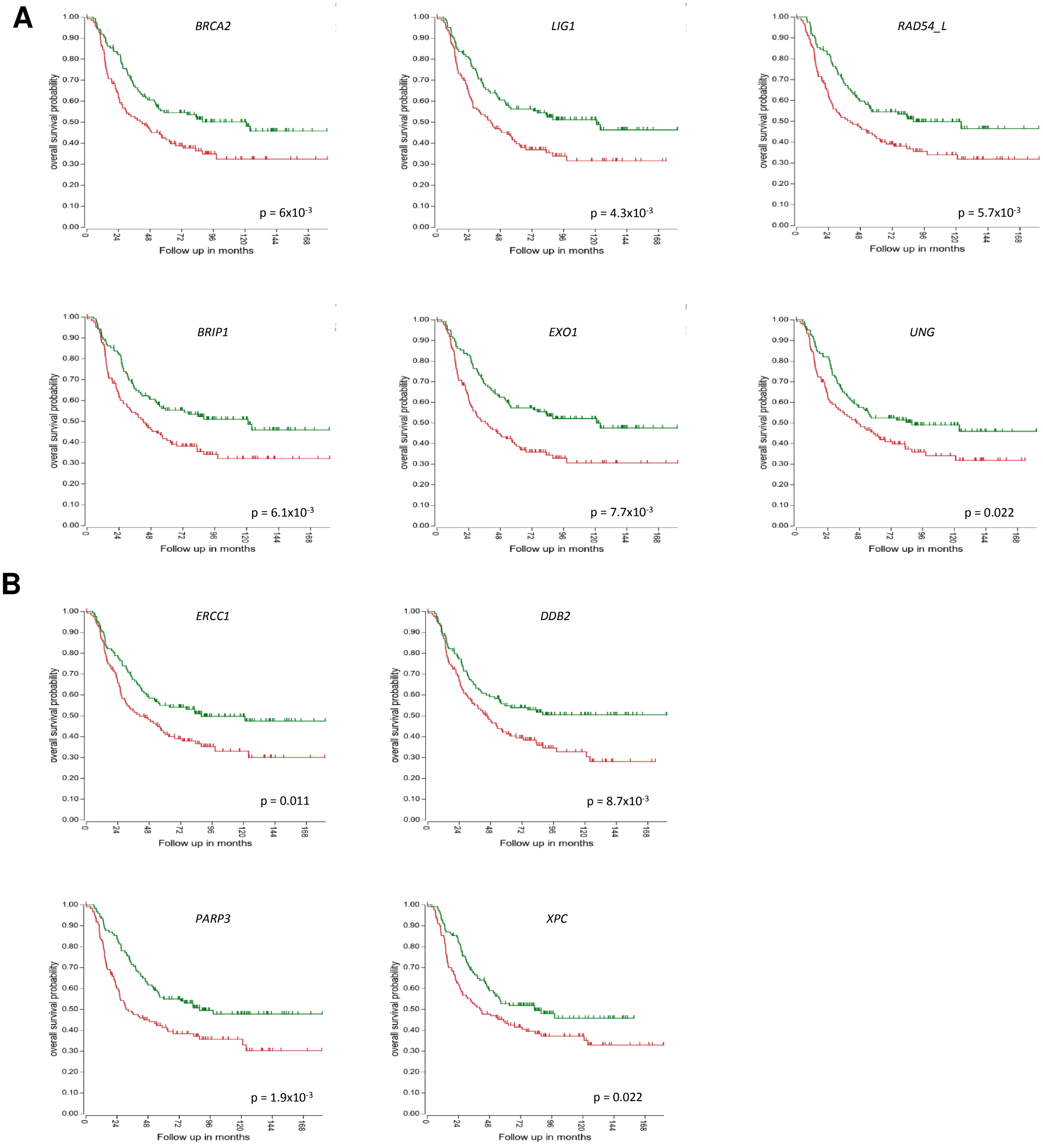

Supplementary Figure 5

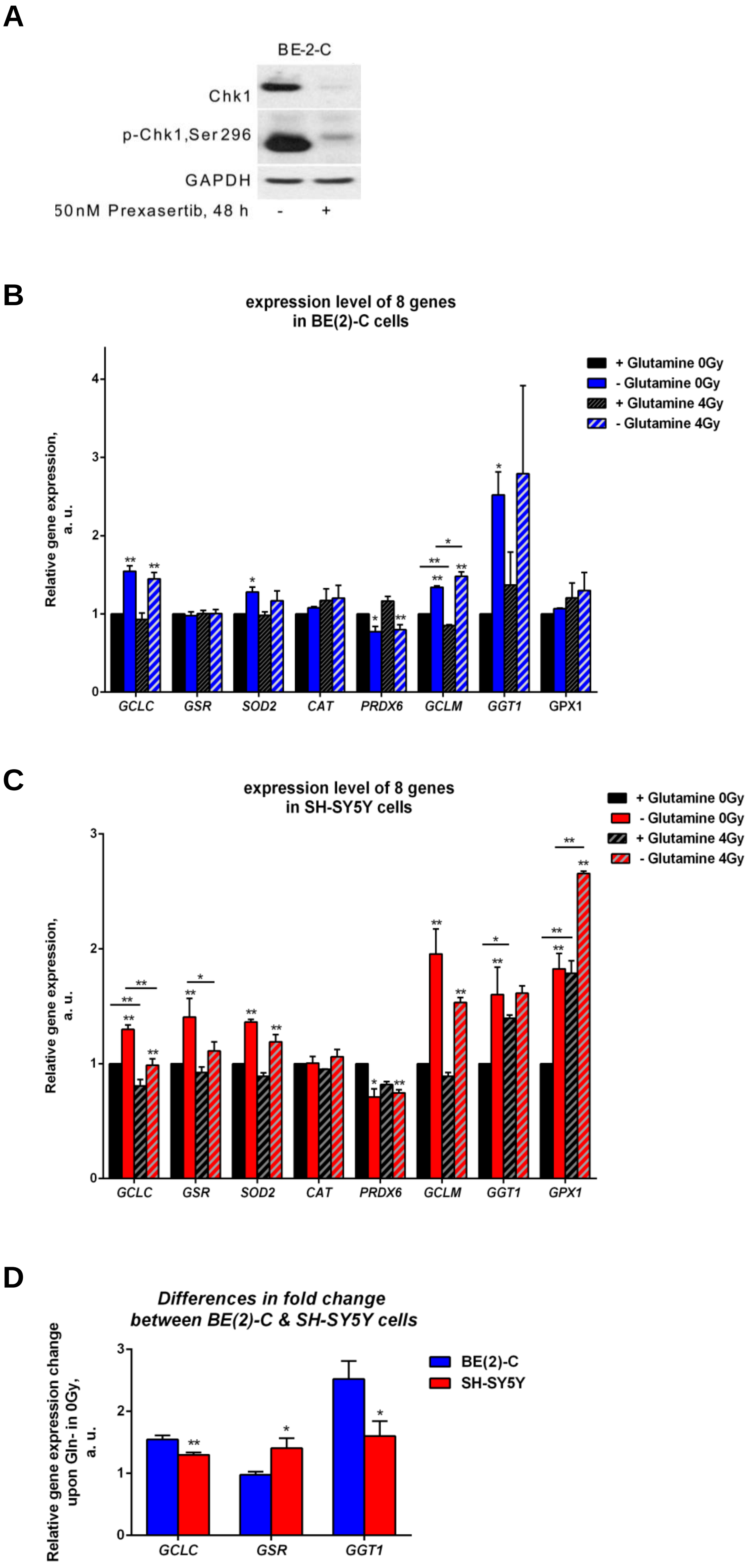

Supplementary Figure 6

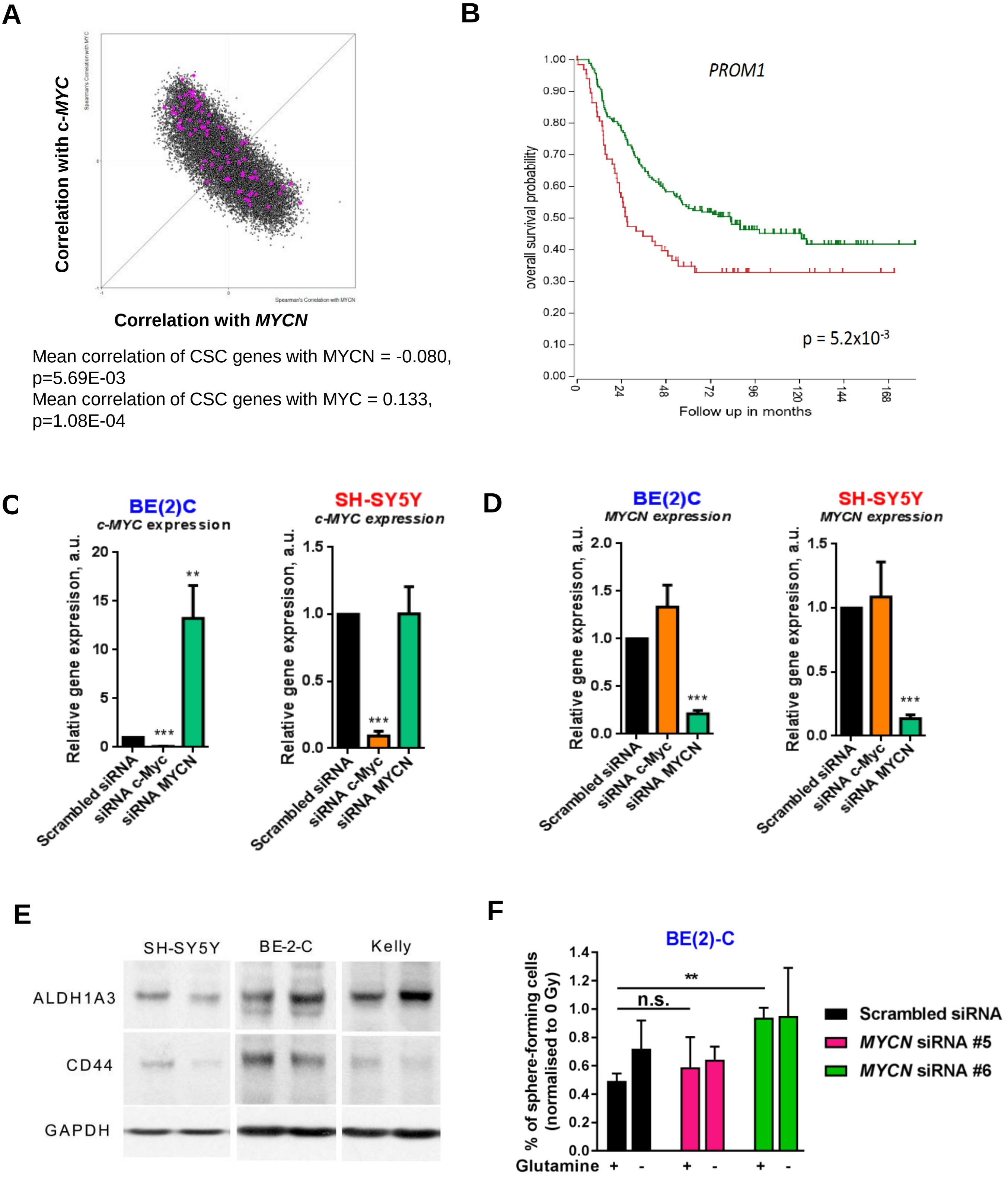

Supplementary Figure 7

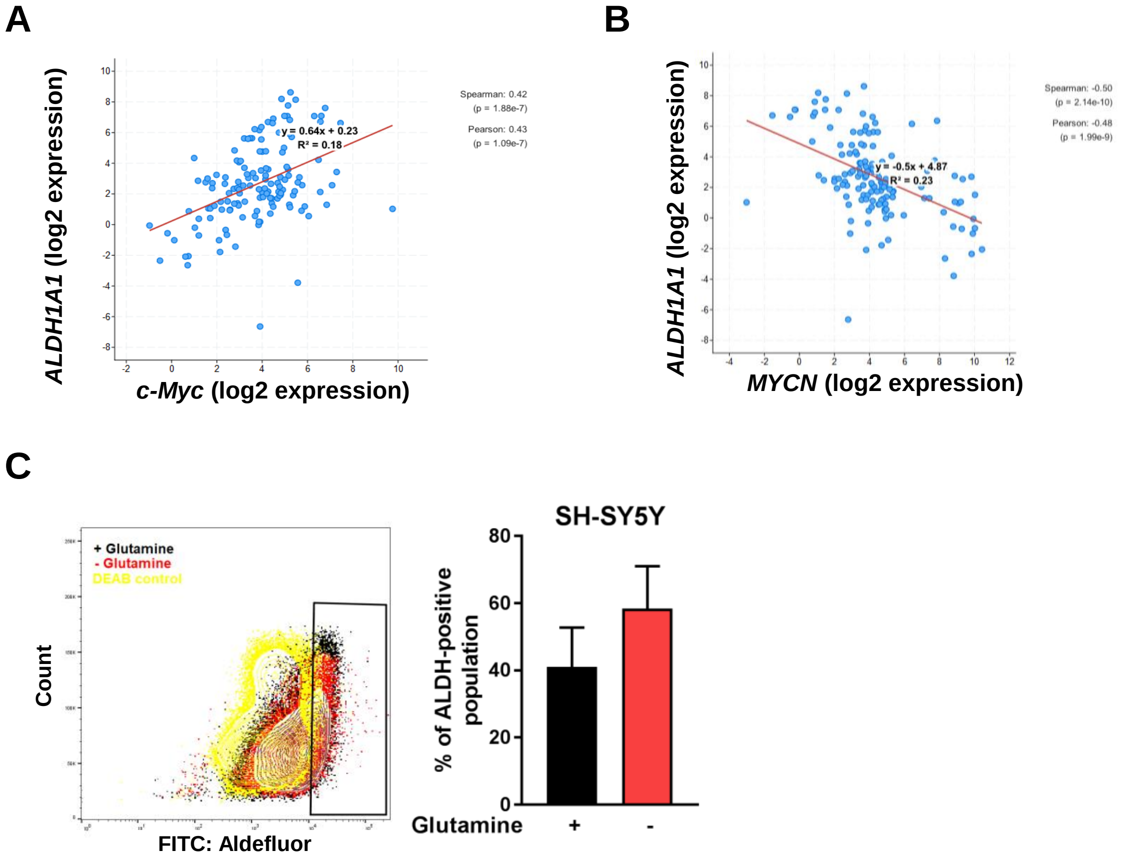

Supplementary Figure 8
